# Synthesis of new fluorescent molecules having an aggregation-induced emission property derived from 4-fluoroisoxazoles

**DOI:** 10.3762/bjoc.16.117

**Published:** 2020-06-22

**Authors:** Kazuyuki Sato, Akira Kawasaki, Yukiko Karuo, Atsushi Tarui, Kentaro Kawai, Masaaki Omote

**Affiliations:** 1 Faculty of Pharmaceutical Sciences, Setsunan University, 45-1 Nagaotoge-cho, Hirakata, Osaka 573-0101, Japan

**Keywords:** aggregation-induced emission, boron ketoiminates, fluorescent probe, α-fluorinated boron ketoiminates, 4-fluoroisoxazoles

## Abstract

Fluorescent molecules based on a fluorinated isoxazole scaffold were synthesized and investigated for their photochemical properties. The introduction of a fluorine substituent into 3,5-diarylisoxazoles led to an increase of fluorescence intensity and exhibited a redshift in the emission intensity. α-Fluorinated boron ketoiminates (F-BKIs) were also synthesized via a ring-opening reaction of 4-fluoroisoxazoles and exhibited highly fluorescent luminescence and aggregation-induced emission (AIE), showing promise as a new fluorophore.

## Introduction

Fluorescence bioprobes based on conventional organic dyes are used for enzyme activity measurements and in bioimaging systems with promising applications in the field of clinical diagnostics [1–7]. Most of the fluorescence bioprobes are mainly excited with near-ultraviolet or blue light ray and the structures often include fluorescein, rhodamine, or 7-amino-4-methylcoumarin (7-AMC) scaffolds as fluorophores. These fluorophores usually exhibit strong fluorescence in dilute solutions, but most of their emissions are partially or completely quenched in the solid state or in highly concentrated solutions by aggregation-caused quenching (ACQ) [8]. On the other hand, there are molecules that exhibit strong emission even in poor solvents or in the solid state. This property is referred to as aggregation-induced emission (AIE) and has attracted much attention in the field of fluorescence bioprobes [9–14]. For example, it is presumed that prion disease, which is caused by the accumulation of prion protein aggregates in the brain, plays an important role in the pathophysiological mechanism of prion protein-polymerized oligomers. However, since prion protein oligomers cannot be visualized using fluorescent probes, the use of AIE fluorescent probes is being investigated as a tool for analyzing the causal relationship between prion diseases and prion proteins.

**Scheme 1:**
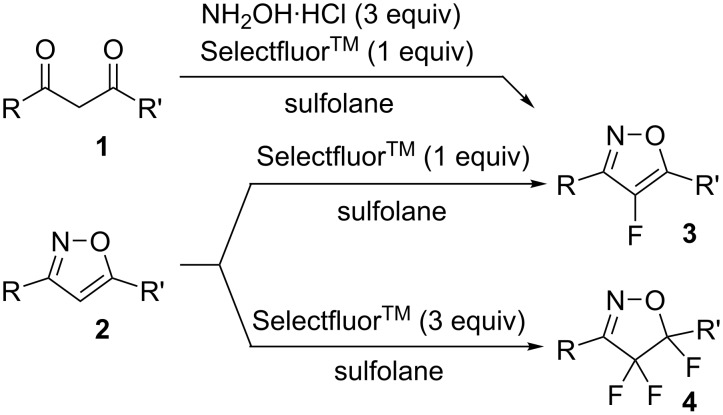
Selective fluorination of isoxazoles and one-pot synthesis of 4-fluoroisoxazoles.

The importance of fluorinated heterocyclic derivatives in the pharmaceutical and agrochemical industries continues to grow, with several fluorinated 6-membered heteroaromatic derivatives finding applications in a wide variety of drugs and plant-protective agents [15–27]. However, there are only a few reports on the synthesis and properties of fluorinated 5-membered heteroaromatic systems, especially those comprising two heteroatoms such as pyrazoles [28–29], isoxazoles [30], and thiazoles [31–32]. Recently, we reported the selective fluorination of isoxazoles, to give monofluorinated isoxazoles **3** or trifluorinated isoxazolines **4** in moderate to good yields (Scheme 1) [33]. In addition, we reported that the reaction proceeded smoothly by starting with 1,3-diketones (**1**) to give **3** in excellent yields in a one-pot reaction.

As part of a wider research program aimed at the applications of fluorinated 5-membered heteroaromatic systems, in this paper, we report the fluorescent luminescence characteristics of 4-fluoroisoxazoles, the synthesis of α-fluorinated boron ketoiminates (F-BKIs), and their photochemical properties.

## Results and Discussion

### Synthesis and optical properties of 4-fluorinated isoxazoles

**Scheme 2:**
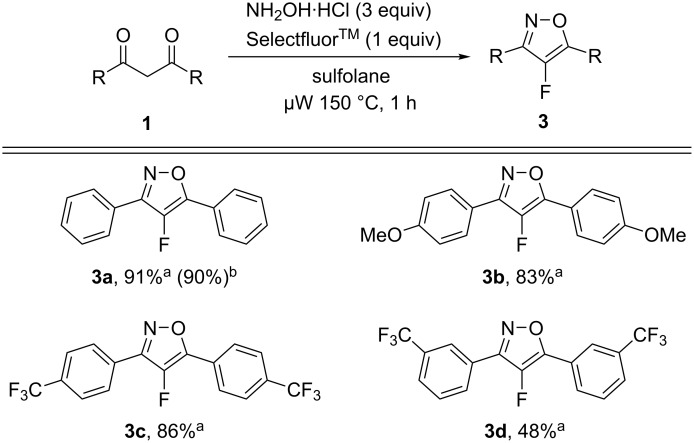
One-pot reaction for the synthesis of 3,5-disubstituted 4-fluoroisoxazoles **3**. ^a^Isolated yield. ^b^Isolated yield by using conventional heating (oil bath) at 150 °C for 1 h.

Although there is a large number of fluorescent molecules, fluorescent probes having an isoxazole scaffold are rare and the limited examples that are available also contain other fluorophores such as styryl, anthranyl, or pyrenyl groups in the molecules. We recently reported the synthesis of 3,5-diaryl-4-fluoroisoxazoles **3** that were found to have planar structures suggesting that they might have the potential to act as a fluorophore [33]. During the synthesis of 3,5-diaryl 4-fluoroisoxazoles **3** according to the previous method (Scheme 2), we noted that 3,5-bis(4-methoxyphenyl)-4-fluoroisoxazole (**3b**) and 3,5-bis(4-trifluoromethylphenyl)-4-fluoroisoxazole (**3c**) exhibited fluorescent properties by irradiation with a UV lamp.

**Figure 1:**
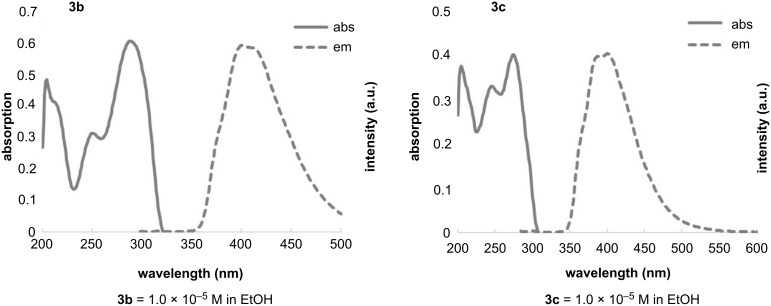
UV–vis and fluorescence (FL) spectra of compounds **3b** and **3c**.

Among the non-fluorinated isoxazoles, only **2c** demonstrated fluorescent emission, although it was very weak. Thus, we decided to further investigate the photochemical properties and the results were summarized in Figure 1 and Table 1. Introducing a fluorine substituent into the isoxazole scaffold led to an increasing fluorescent intensity and exhibited a redshift in the emission intensity. Interestingly, the excitation maximum of **3** showed a redshift of approximately 20 nm with the incorporation of a single fluorine atom into the isoxazole scaffold in comparison with **2c**. This observation suggested that the strong electronegativity of fluorine might affect the electron density on the isoxazole ring.

**Table 1:** UV–vis absorption and fluorescence data for 4-fluoroisoxazoles **3b** and **3c**, and non-fluorinated compound **2c**^a^.

dye	λ_abs_ (nm)	ε (M^−1^·cm^−1^)	λ_em_ (nm)	Stokes shift (cm^−1^)^b^

**3b**	288	60476	400	9722
**3c**	274	40251	400	11496
**2c**	270	43015	370	10010

^a^Measurement conditions: 1.0 × 10^−5^ M in EtOH, excitation at λ = 288 nm for **3b**, 274 nm for **3c**, and 270 nm for **2c**. ^b^Stokes shift = 1/λ_ex_ − 1/λ_em_ (cm^−1^).

### Synthesis of boron ketoiminates and α-fluorinated boron ketoiminates

**Scheme 3:**
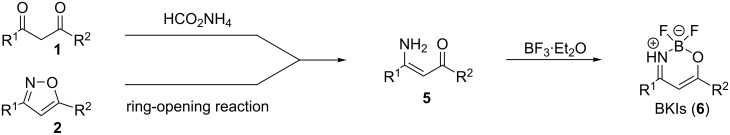
Synthesis of BKIs **6** either from 1,3-diketones **1** or from isoxazoles **2**.

Boron ketoiminates (BKIs, **6**) are one of the new types of boron-chelating dye [34–38], their optical properties feature a large Stokes shift and high molar absorption coefficients (ε) that are similar to the corresponding boron diketonates. The synthesis and properties of BKIs have been reported recently and they are easily accessible either from the corresponding 1,3-diketones **1** or from isoxazoles **2** through a ring-opening reaction (Scheme 3).

**Scheme 4:**
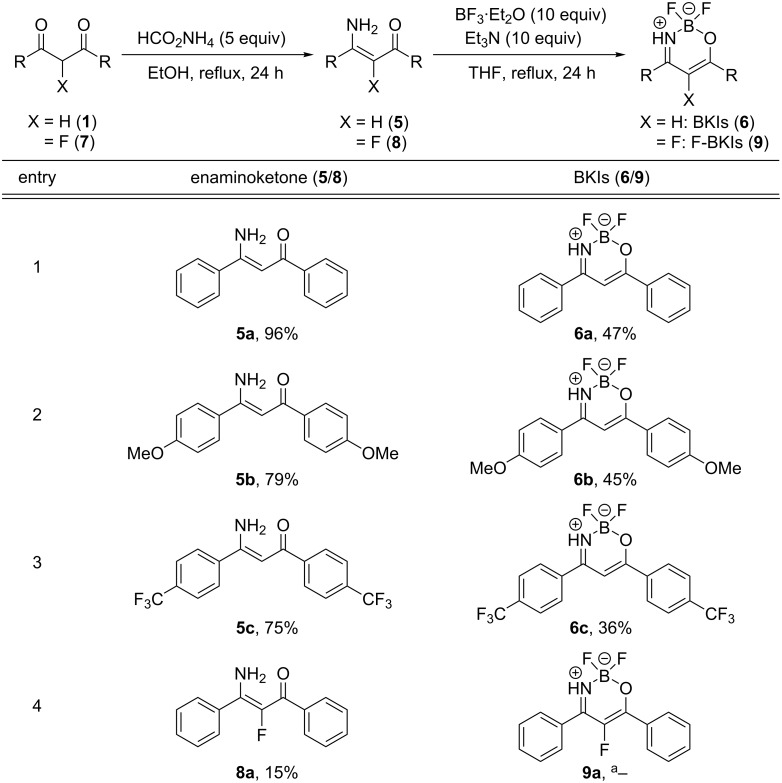
Synthesis of enaminoketones **5** and **8** and their conversion to BKIs (yields refer to isolated yields; ^a^boron complexation of **8a** to **9a** was not attempted).

Based on the above observations, we attempted to introduce a fluorine atom into BKIs to access the corresponding α-fluorinated boron ketoiminates (F-BKIs, **9**). First, we started from 1,3-diketones **1** and reacted them with ammonium formate to give the corresponding enaminoketones **5** in high yields (see entries 1–3 in Scheme 4). Then, compounds **5** were treated with 10 equiv of BF_3_·Et_2_O in anhydrous THF solution in the presence of an excess of Et_3_N to give BKIs **6** in moderate yields. However, when the same conditions were applied to the fluorinated diketone, 2-fluoro-1,3-diphenylpropane-1,3-dione (**7a**), the corresponding enaminoketone **8a** was obtained in only low yield (Scheme 4, entry 4) and we did not attempt the conversion of **8a** towards the α-fluorinated boron ketoiminate **9a**.

**Scheme 5:**
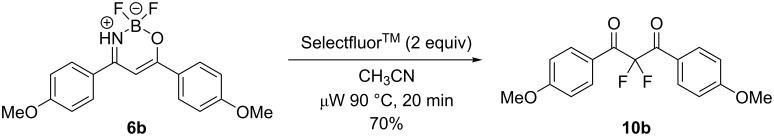
Attempted selective fluorination of BKI **6b**.

Next, we attempted the selective fluorination of **6b** to obtain the desired fluorinated analog **9b**. However, in the synthesis of F-BKIs through the selective fluorination of the corresponding BKIs, the use of 1 equiv of Selectfluor did not give any product and performing the reaction with excess amounts of Selectfluor gave rise to the corresponding α,α-difluorinated diketone (Scheme 5).

**Scheme 6:**
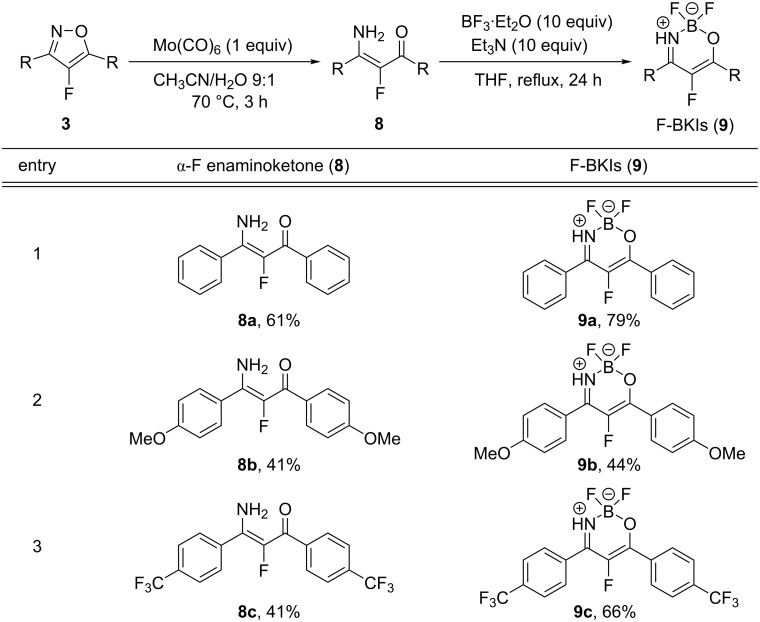
Ring-opening reaction of 4-fluoroisoxazoles **3** and their conversion into F-BKIs **9** (yields refer to isolated yields).

As an alternative method to synthesize F-BKIs **9**, we turned our attention to the ring-opening reaction of isoxazoles. The reductive cleavage of the N–O bond in isoxazoles can be achieved by transition metals or their complexes to give the corresponding enaminoketones [35,37]. Consequently, we examined several conditions for the ring opening of fluorinated isoxazoles **3**, and found that using Mo(CO)_6_ gave the corresponding α-fluorinated enaminoketones **8** in moderate yields (Scheme 6). With the enaminoketones **8** at hand, the subsequent boron complexation with BF_3_·Et_2_O in the presence of Et_3_N gave the desired F-BKIs **9** in moderate to good yields.

### Optical properties of boron ketoiminates and α-fluorinated boron ketoiminates

**Figure 2:**
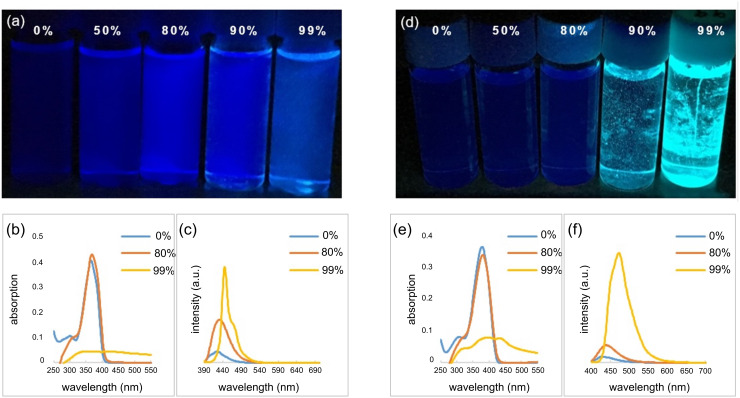
Photochemical properties comparisons of BKIs and F-BKIs. (a–c) BKI **6b**: photograph (a), UV–vis (b), and FL (c) spectra at different solvent compositions of THF/H_2_O upon excitation at 365 nm (1.0 × 10^−5^ M); (d–f) F-BKI **9b**: photograph (d), UV–vis (e), and FL (f) spectra at different solvent compositions of THF/H_2_O upon excitation at 380 nm (1.0 × 10^−5^ M).

Chujo and co-workers described that BKIs could be a promising structural motif for having AIE properties [36]. For the purpose of comparison with the photochemical properties of BKIs and F-BKIs, we measured the optical properties of compounds **6** and **9** (Table 2). As shown in Figure 2, the UV–vis absorptions of **6b** and **9b** in THF decreased upon the addition of H_2_O, and white precipitates formed in samples exceeding 80% of water content. Concurrently, the fluorescent luminescence (FL) of the solutions of **6b** and **9b** exhibited an increase in the emission intensities with increasing water content. It was interesting to note that the excitation maximum of **9b** in the aggregated state showed a red-shift by approximately 20 nm based on the incorporation of a single fluorine atom into the boron ketoiminate scaffold in comparison with **6b**. On the other hand, unfortunately, no similar behavior could be observed for the other F-BKIs. This effect of **9b** bearing OCH_3_ groups on both benzene rings might be attributed to the energy gap between HOMO and LUMO based on the electron-density distribution of boron ketoiminate scaffold induced by the strong electronegative fluorine atom [39]. The FL intensities were lower than that of the corresponding BKIs, a similar tendency to what was also observed in other F-BKIs **9a** and **9c**. In summary, we found that the F-BKIs described in this report exhibited AIE behavior.

**Table 2:** Optical properties of BKIs and F-BKIs.

dye	λ_abs_ (nm)^a^	λ_ex(agg)_ (nm)^b^	λ_em(agg)_ (nm)^b^	Stokes shift_(agg)_ (cm^−1^)^c^

**6a**	351	352	478	4489
**6b**	365	365	452	5274
**6c**	353	332	517	10778
**9a**	362	365	446	4976
**9b**	378	380	472	5129
**9c**	361	366	494	7080

^a^Measured in THF solution (1.0 × 10^−5^ M). ^b^Measured in THF/H_2_O 1:99 mixed solvent (1.0 × 10^−5^ M). ^c^Stokes shift = 1/λ_ex(agg)_ − 1/λ_em(agg)_ (cm^−1^).

## Conclusion

In conclusion, we demonstrated that 3,5-diaryl-4-fluoroisoxazoles exhibited fluorescent luminescence, although, the emissions were not strong. Interestingly the introduction of a fluorine substituent into the isoxazole scaffold led to an increase in the fluorescent intensity in the aggregated state and exhibited a redshift in the emission intensity. We also achieved the first synthesis of α-fluorinated boron ketoiminates (F-BKIs) by the reductive cleavage of the N–O bond in 4-fluorinated isoxazoles and demonstrated that F-BKIs exhibited AIE property similarly to their parent BKI. Further structural modifications of compounds **3** or **9** and applications to fluorescent bioprobes are currently under investigation.

## Supporting Information

File 1 General procedures and analytical data, including copies of ^1^H NMR, ^13^C NMR and ^19^F NMR spectra.
