# Supplementary material for: Synthesis of new fluorescent molecules having an aggregation-induced emission property derived from 4-fluoroisoxazoles
Source: Beilstein J Org Chem. 2020 Jun 22;16:1411–7. doi: 10.3762/bjoc.16.117 (PMC7323617; doi:10.3762/bjoc.16.117)
Supplement: File 1 — General procedures and analytical data, including copies of 1H NMR, 13C NMR and 19F NMR spectra. [file Beilstein_J_Org_Chem-16-1411-s001.pdf]

BEILSTEIN JOURNAL OF ORGANIC CHEMISTRY

Supporting Information

for

Synthesis of new fluorescent molecules having an aggregation-induced emission property derived from 4-fluoroisoxazoles

Kazuyuki Sato, Akira Kawasaki, Yukiko Karuo, Atsushi Tarui, Kentaro Kawai and Masaaki Omote

Beilstein J. Org. Chem. **2020,** *16,* 1411–1417. doi:10.3762/bjoc.16.117

General procedures and analytical data, including copies of ^{1}H NMR, ^{13}C NMR and ^{19}F NMR spectra

License and Terms: This is a supporting information file under the terms of the Creative Commons Attribution License (http://creativecommons.org/licenses/by/4.0). Please note that the reuse, redistribution and reproduction in particular requires that the authors and source are credited.
The license is subject to the *Beilstein Journal of Organic Chemistry* terms and conditions: (https://www.beilstein-journals.org/bjoc)

Table of contents

General information ……………………………………………… S2
Materials ……………………………………………………… S2
Experimental section
General procedure for the synthesis of
4-fluoroisoxazoles (**3**). …………….. S2
General procedure for the synthesis of
boron ketoiminates (**6**). …………..…S3
General procedure for the synthesis of
α-fluorinated boron ketoiminates (F-BKIs, **9**). …S3
Spectroscopic data ………………………………………….. S4–S7
References ……………………………..……………………... S7
UV–vis and FL spectra …………………………...……. S8–S9
NMR spectra ……………………………………...…. S10–S23

General Information:

^{1}H NMR and ^{13}C NMR spectra were recorded on JEOL JNM-ECZS-400 and ECA-600SN spectrometers. ^{19}F NMR spectra were recorded on JEOL JNM-ECZS-400 spectrometer. Chemical shifts of ^{1}H NMR are reported in ppm from tetramethylsilane (TMS: 0 ppm) as an internal standard. Chemical shifts of ^{13}C NMR are reported in ppm from tetramethylsilane (TMS: 0 ppm) or from the solvent (DMSO-d_6: 39.520 ppm) as an internal standard. Chemical shifts of ^{19}F NMR are reported in ppm from trichlorofluoromethane ($CFCl_3$: 0 ppm) as an internal standard. All data are reported as follows: chemical shifts, relative integration value, multiplicity (s = singlet, d = doublet, t = triplet, q = quartet, br = broad, m = multiplet), coupling constants (Hz). Mass spectra were obtained on JEOL JMS-700T spectrometers. Melting points were measured on Yanagimoto micro melting point apparatus MP-S3. UV–vis absorption spectra were measured on a Jasco V-650 spectrometer. Fluorescence spectra were measured on a Jasco FP-8600 spectrofluorometer and appropriate blank spectra were subtracted. Microwave reactions were performed in microwave tubes with clip lids using Biotage Initiator+ microwave reactor.

Materials:

Solvents (sulfolane, CH_3CN and EtOH) were distilled under inert argon atmosphere or under reduced pressure before use. Tetrahydrofuran (THF) was purchased from Kanto Chemical Co. Inc. as “Dehydrated”. All commercially available reagents were used without further purification. All experiments were carried out under argon atmosphere unless otherwise noted.

Experimental section:

General procedure for the synthesis of 4-fluoroisoxazoles 3 [1].

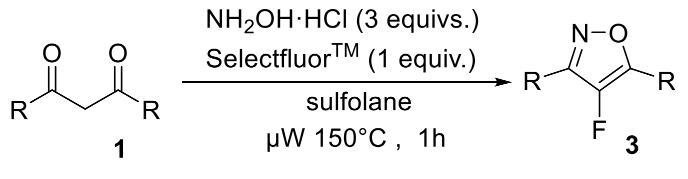

1,3-Diketone (**1**; 1 mmol), hydroxylamine hydrochloride (3 mmol) and Selectfluor^{TM} (1 mmol) were added to a microwave vial and suspended in sulfolane (4 mL). The vial was sealed and heated by microwave irradiation for 1 hour at 150 °C. The resulting mixture was quenched with $NaHCO_3$ and extracted with AcOEt. The AcOEt layer was washed with sat. NaCl and dried over $MgSO_4$. The solvent was removed in vacuo and the residue purified by column chromatography to give the 4-fluoroisoxazoles **3**.

General procedure for the synthesis of boron ketoiminates 6.

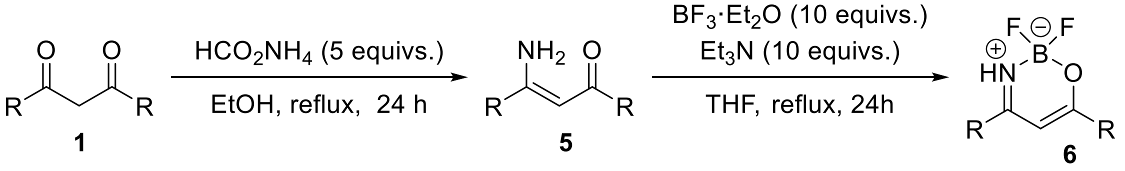

The mixture of 1,3-diketone (**3**, 1 mmol) and ammonium formate (5 mmol) in ethanol (5.5 mL) was stirred under reflux condition for 24 hours, and then the ethanol was distilled off under reduced pressure. To the residue was added AcOEt and water, and extracted. The AcOEt layer was dried over $MgSO_4$ and the solvent was removed in vacuo. The residue was passed through a silica gel column to give the enaminoketone (**5**), the formation of which was checked by NMR.

To a solution of the enaminoketone (**5**, 1 mmol) in THF (14 mL) was added $BF_3{\cdot}Et_2O$ (10 mmol) and triethylamine (10 mmol), and the mixture was heated under reflux for 24 hours. The resulting mixture was quenched with H_2O and extracted with AcOEt. The AcOEt layer was washed with sat. NaCl and dried over $MgSO_4$. The solvent was removed in vacuo and the residue was purified by column chromatography to give the boron ketoiminates (BKIs, **6**).

General procedure for the synthesis of α-fluorinated boron ketoiminates (F-BKIs, 9).

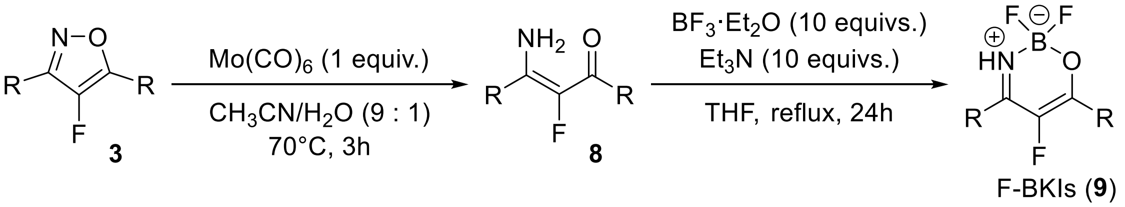

The solution of the appropriate 4-fluoroisoxazole **3** (1 mmol) in CH_3CN/H_2O 9:1 (27 mL) was stirred for 15 min at room temperature. Then, $Mo(CO)_6$ (1.1 mmol) was added into the solution, and the mixture was heated at 70 °C for 3 hours. The resulting mixture was quenched with sat. NaCl and extracted with AcOEt. The AcOEt layer was dried over $MgSO_4$ and the solvent was removed in vacuo. The residue was passed through a silica gel column for separation of the corresponding defluorinated enaminoketone **5** and the α-monofluoro-1,3-diketone to give the desired α-fluorinated enaminoketone **8**, which was checked by NMR for the formation.

To a solution of the α-fluorinated enaminoketone **8** (1 mmol) in THF (14 mL) was added $BF_3{\cdot}Et_2O$ (10 mmol) and triethylamine (10 mmol), and the mixture was heated under reflux condition for 24 hours. The resulting mixture was quenched with H_2O and extracted with AcOEt. The AcOEt layer was washed with sat. NaCl and dried over $MgSO_4$. The solvent was removed in vacuo and the residue was purified by column chromatography to give the α-fluorinated boron ketoiminate (F-BKIs, **9**).

Spectroscopic data:

4-Fluoro-3,5-bis(3-(trifluoromethyl)phenyl)isoxazole (3d)

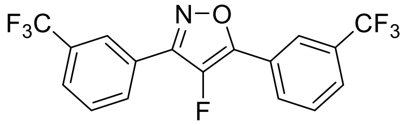

Colorless solid; M.p. 96.5–97.5 °C; ^{1}H NMR (400 MHz, $CDCl_3$) δ: 7.66–7.81 (4H, m), 8.06–8.23 (4H, m); ^{13}C NMR (100 MHz, $CDCl_3$) δ: 122.11 (m), 123.59 (q, *J* = 272.6 Hz), 123.69 (q, *J* = 272.6 Hz), 123.92 (m), 126.25 (d, *J* = 5.2 Hz), 126.96 (m), 127.03 (d, *J* = 4.0 Hz), 127.39 (m), 129.33 (d, *J* = 4.9 Hz), 129.74, 129.87, 130.20, 131.69 (q, *J* = 32.8 Hz), 131.83 (q, *J* = 33.0 Hz), 141.92 (d, *J* = 261.0 Hz), 152.28, (d, *J* = 10.5 Hz), 152.70 (d, *J* = 19.1 Hz); ^{19}F NMR (376 MHz, $CDCl_3$) δ: -62.78 (3F, s), -62.89 (3F, s), -175.83 (1F, s); MS *m/z*: 375 (M^+); HRMS Calcd for $C_{17}H_8F_7NO$: 375.0494 (M^+), Found: 375.0497.

3-Amino-1,3-diphenylprop-2-en-1-one (5a) [2]

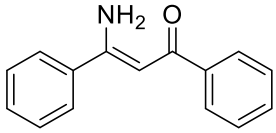

^{1}H NMR (400 MHz, $CDCl_3$) δ: 5.45 (1H, bs), 6.15 (1H, s), 7.41–7.53 (6H, m), 7.63–7.66 (2H, m), 7.94–7.97 (2H, m), 10.42 (1H, bs).

3-Amino-1,3-bis(4-methoxyphenyl)prop-2-en-1-one (5b) [2]

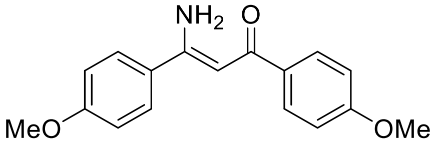

^{1}H NMR (400 MHz, $CDCl_3$) δ: 3.86 (3H, s), 3.87 (3H, s), 5.33 (1H, bs), 6.10 (1H, s), 6.92–7.00 (4H, m), 7.58–7.61 (2H, m), 7.92–7.96 (2H, m), 10.41 (1H, bs).

3-Amino-1,3-bis(4-(trifluoromethyl)phenyl)prop-2-en-1-one (5c) [2]

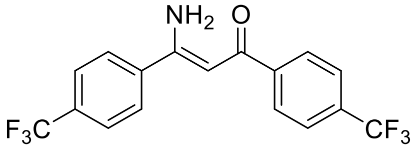

^{1}H NMR (400 MHz, $CDCl_3$) δ: 5.55 (1H, bs), 6.11 (1H, s), 7.69–7.71 (2H, m), 7.76 (4H, s), 8.01–8.03 (2H, m), 10.42 (1H, bs); ^{19}F NMR (376 MHz, $CDCl_3$) δ: -62.70 (3F, s), -62.80 (3F, s).

2,2-Difluoro-4,6-diphenyl-2*H*-1,3,2λ^4-oxazaborinine (6a) [3]

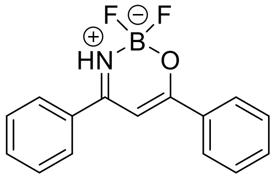

Colorless solid; M.p. 204.5–206.5 °C; ^{1}H NMR (400 MHz, DMSO-d6) δ: 6.93 (1H, d, *J* = 1.8 Hz), 7.53–7.73 (6H, m), 8.04–8.14 (4H, m), 10.33 (1H, bs); ^{13}C NMR (100 MHz, DMSO-d6) δ: 92.08, 127.17, 127.92, 128.73, 128.93, 132.30, 132.77, 132.91, 133.33, 169.40, 170.70; ^{19}F NMR (376 MHz, DMSO-d6) δ: -128.03—128.15 (2F, m); MS *m/z*: 271 (M^+); HRMS Calcd for $C_{15}H_{12}BF_2NO$: 271.0980 (M^+), Found: 271.0982.

2,2-Difluoro-4,6-bis(4-methoxyphenyl)-2*H*-1,3,2λ^4-oxazaborinine (6b) [4]

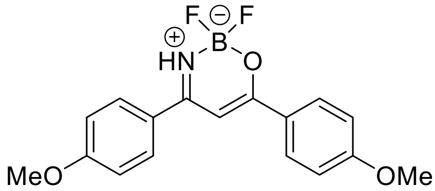

Pale yellow solid; M.p. 220.0–223.0 °C; ^{1}H NMR (400 MHz, DMSO-d6) δ: 3.86 (3H, s), 3.88 (3H, s), 6.82 (1H, d, *J* = 1.3 Hz), 7.07–7.14 (4H, m), 8.08–8.10 (4H, m), 9.85 (1H, bs); ^{13}C NMR (100 MHz, DMSO-d6) δ: 55.42, 55.55, 90.20, 114.11, 114.31, 124.71, 125.78, 129.18, 129.97, 162.55, 163.05, 167.56, 170.02; ^{19}F NMR (376 MHz, DMSO-d6) δ: -128.87—128.95 (2F, m); MS *m/z*: 331 (M^+); HRMS Calcd for $C_{17}H_{16}BF_2NO_3$: 331.1191 (M^+), Found: 331.1193.

2,2-Difluoro-4,6-bis(4-(trifluoromethyl)phenyl)-2*H*-1,3,2λ^4-oxazaborinine (6c)

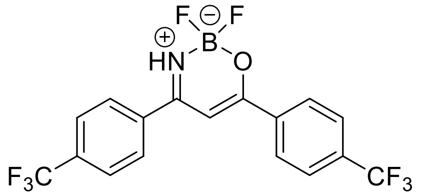

Pale yellow solid; M.p. 265.0–270.0 °C; ^{1}H NMR (400 MHz, DMSO-d6) δ: 7.10 (1H, d, *J* = 1.4 Hz), 7.91–8.00 (4H, m), 8.23–8.35 (4H, m), 10.82 (1H, bs); ^{13}C NMR (100 MHz, DMSO-d6) δ: 93.86, 123.58 (q, *J* = 272.6 Hz), 123.72 (q, *J* = 272.6 Hz), 125.67 (q, *J* = 4.1 Hz), 125.75 (q, *J* = 4.1 Hz), 128.05, 129.12, 132.06 (q, *J* = 31.9 Hz), 132.38 (q, *J* = 31.9 Hz), 136.46, 136.94, 169.01, 169.26; ^{19}F NMR (376 MHz, DMSO-d6) δ: -61.44 (3F, s), -61.55 (3F, s), -127.35—127.42 (2F, m); MS *m/z*: 407 (M^+); HRMS Calcd for $C_{17}H_{10}BF_8NO$: 407.0728 (M^+), Found: 407.0724.

3-Amino-2-fluoro-1,3-diphenylprop-2-en-1-one (8a)

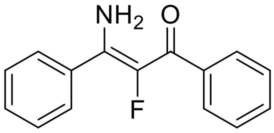

^{1}H NMR (400 MHz, $CDCl_3$) δ: 7.42–7.51 (6H, m), 7.63–7.65 (2H, m), 7.89–7.92 (2H, m); ^{19}F NMR (376 MHz, $CDCl_3$) δ: -170.44 (1F, s).

3-Amino-2-fluoro-1,3-bis(4-methoxyphenyl)prop-2-en-1-one (8b)

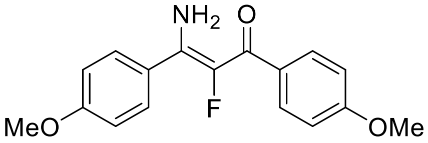

^{1}H NMR (400 MHz, $CDCl_3$) δ: 3.86 (3H, s), 3.86 (3H, s), 6.92–7.01 (4H, m), 7.58–7.62 (2H, m), 7.93–7.97 (2H, m); ^{19}F NMR (376 MHz, $CDCl_3$) δ: -169.97 (1F, s).

3-Amino-2-fluoro-1,3-bis(4-(trifluoromethyl)phenyl)prop-2-en-1-one (8c)

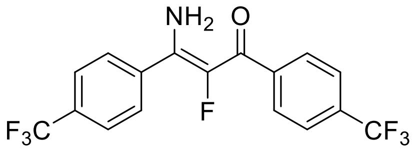

^{1}H NMR (400 MHz, $CDCl_3$) δ: 7.70–7.73 (2H, m), 7.77 (4H, s), 7.99–8.01 (2H, m); ^{19}F NMR (376 MHz, $CDCl_3$) δ: -62.83 (3F, s), -62.86 (3F, s), -170.60 (1F, s).

2,2,5-Trifluoro-4,6-diphenyl-2*H*-1,3,2λ^4-oxazaborinine (9a)

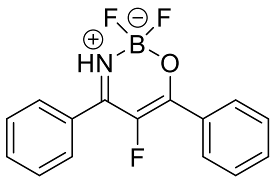

Pale yellow solid; M.p. 172.5–174.0 °C; ^{1}H NMR (400 MHz, DMSO-d6) δ: 7.57–7.73 (6H, m), 7.79–7.81 (2H, m), 7.96–7.98 (2H, m), 10.80 (1H, bs); ^{13}C NMR (100 MHz, DMSO-d6) δ: 128.49 (d, J = 8.3 Hz), 128.63, 128.75, 129.05 (d, J = 3.8 Hz), 129.34, 130.79 (d, J = 5.7 Hz), 132.16, 132.61, 139.59 (d, J = 221.5 Hz), 157.0 (d, J = 20.8 Hz), 165.24 (d, J = 26.1 Hz); ^{19}F NMR (376 MHz, DMSO-d6) δ: -129.38—129.50 (2F, m), -171.41 (1F, s); MS *m/z*: 289 (M^+); HRMS Calcd for $C_{15}H_{11}BF_3NO$: 289.0886 (M^+), Found: 289.0888.

2,2,5-Trifluoro-4,6-bis(4-methoxyphenyl)-2*H*-1,3,2λ⁴-oxazaborinine (9b)

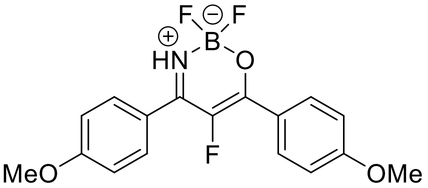

Yellow solid; M.p. 188.0–189.0 °C; ^{1}H NMR (400 MHz, DMSO-d6) δ: 3.87 (3H, s), 3.88 (3H, s), 7.11–7.17 (4H, m), 7.78–7.81 (2H, m), 7.95–7.97 (2H, m), 10.28 (1H, bs); ^{13}C NMR (100 MHz, DMSO-d6) δ: 55.43, 55.54, 114.15, 114.26, 121.50, 123.12 (d, *J* = 5.8 Hz), 130.66 (d, *J* = 9.1 Hz), 131.38 (d, *J* = 4.7 Hz), 139.28 (d, *J* = 220.9 Hz), 156.68 (d, *J* = 20.8 Hz), 162.28, 162.81, 163.42 (d, *J* = 25.1 Hz); ^{19}F NMR (376 MHz, DMSO-d6) δ: -130.66–-130.73 (2F, m), -171.94 (1F, s); MS *m/z*: 349 (M^+); HRMS Calcd for $C_{17}H_{15}BF_3NO_3$: 349.1097 (M^+), Found: 349.1102.

2,2,5-Trifluoro-4,6-bis(4-(trifluoromethyl)phenyl)-2*H*-1,3,2λ⁴-oxazaborinine (9c)

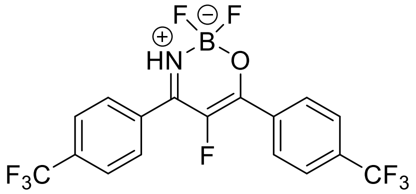

Pale yellow solid; M.p. 224.0–226.0 °C; ^{1}H NMR (400 MHz, DMSO-d6) δ: 7.95–8.05 (6H, m), 8.15–8.17 (2H, m), 11.29 (1H, bs); ^{13}C NMR (100 MHz, DMSO-d6) δ: 123.52 (q, *J* = 272.8 Hz), 123.61 (q, *J* = 272.5 Hz), 125.49 (q, *J* = 3.8 Hz), 125.69 (d, *J* = 3.8 Hz), 129.31 (d, *J* = 8.3 Hz), 130.16 (d, *J* = 3.4 Hz), 131.64 (q, *J* = 32.6 Hz), 132.28 (q, *J* = 32.4 Hz), 132.91, 134.43 (d, *J* = 5.3 Hz), 140.08 (d, *J* = 225.4 Hz), 155.22 (d, *J* = 20.7 Hz), 164.82 (d, *J* = 26.9 Hz); ^{19}F NMR (376 MHz, DMSO-d6) δ: -61.60 (3F, s), -61.63 (3F, s), -128.26–-128.34 (2F, m), -170.12 (1F, s); MS *m/z*: 425 (M^+); HRMS Calcd for $C_{17}H_9BF_9NO$: 425.0633 (M^+), Found: 425.0632.

References:

[1] Sato, K.; Sandford, G.; Shimizu, K.; Akiyama, S.; Lancashire, M. J.; Yufit, D. S.; Tarui, A.; Omote, M.; Kumadaki, I.; Harusawa, S.; Ando, A. *Tetrahedron* **2016**, *72*, 1690–1698.

[2] H. S. P. Rao, N. Muthanna, *Eur. J. Org. Chem.* **2015**, 1525–1532.

[3] I. Bally, E. Ciornei, A. Vasilescu, A. T. Balaban, *Tetrahedron* **1973**, *29*, 3185–3187.

[4] R. Yoshii, A. Nagai, K. Tanaka, Y. Chujo, *Chem. -Eur. J.* **2013**, *19*, 4506–4512.

UV–vis and FL spectra:

2,2-Difluoro-4,6-diphenyl-2*H*-1,3,2λ^4-oxazaborinine (**6a**)

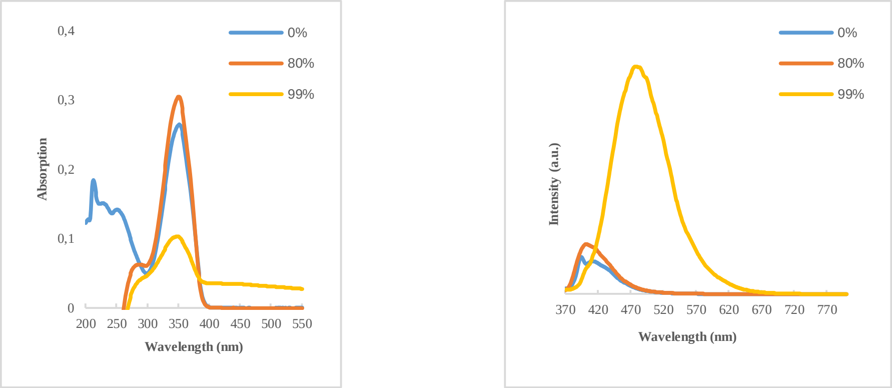

UV/Vis and FL spectra of **6a** with solvent compositions of the THF/H_2O mixture upon excitation at 352 nm (1.0 x 10^{-5} M).

2,2-Difluoro-4,6-bis(4-methoxyphenyl)-2*H*-1,3,2λ^4-oxazaborinine (**6b**)

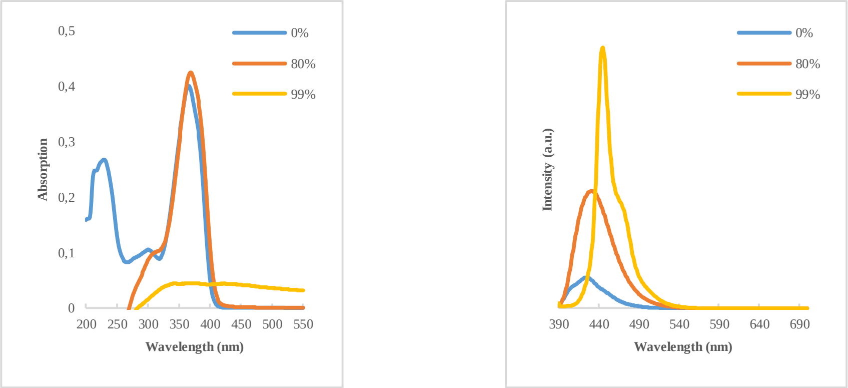

UV/Vis and FL spectra of **6b** with solvent compositions of the THF/H_2O mixture upon excitation at 365 nm (1.0 x 10^{-5} M).

2,2-Difluoro-4,6-bis(4-(trifluoromethyl)phenyl)-2*H*-1,3,2λ^4-oxazaborinine (**6c**)

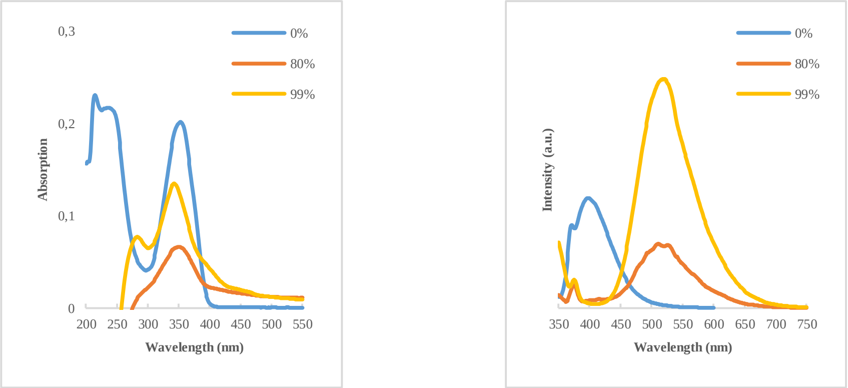

UV/Vis and FL spectra of **6c** with solvent compositions of the THF/H_2O mixture upon excitation at 332 nm (1.0 x 10^{-5} M).

2,2,5-Trifluoro-4,6-diphenyl-2H-1,3,2λ^4-oxazaborinine (**9a**)

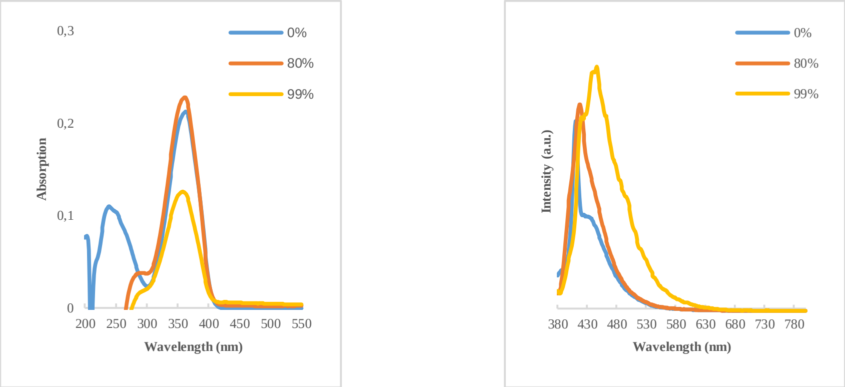

UV/Vis and FL spectra of **9a** with solvent compositions of the THF/H_2O mixture upon excitation at 365 nm (1.0 x 10^{-5} M).

2,2,5-Trifluoro-4,6-bis(4-methoxyphenyl)-2H-1,3,2λ^4-oxazaborinine (**9b**)

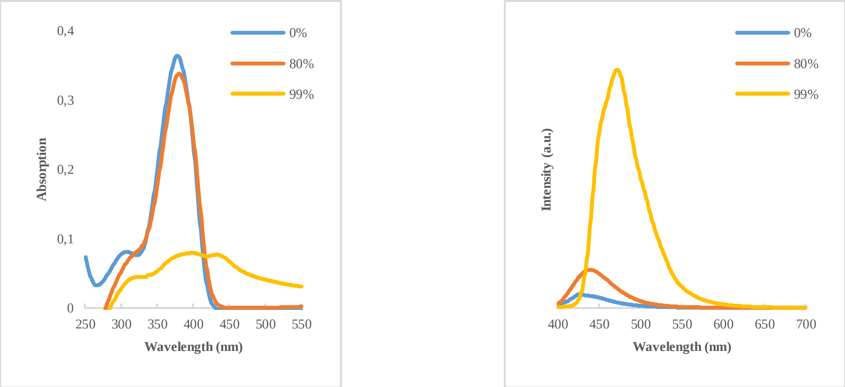

UV/Vis and FL spectra of **9b** with solvent compositions of the THF/H_2O mixture upon excitation at 380 nm (1.0 x 10^{-5} M).

2,2,5-Trifluoro-4,6-bis(4-(trifluoromethyl)phenyl)-2H-1,3,2λ^4-oxazaborinine (**9c**)

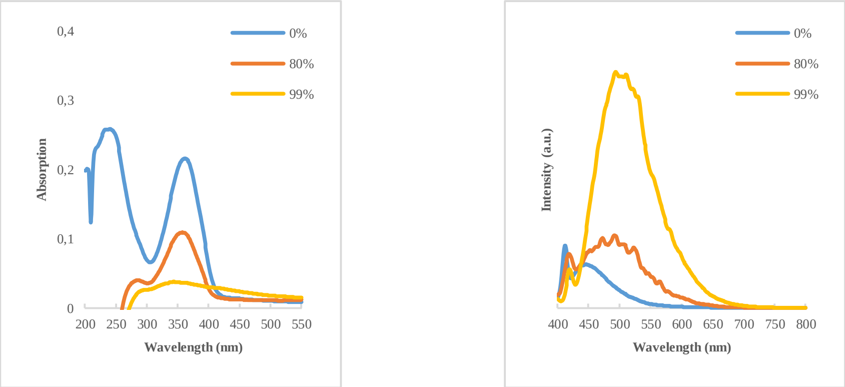

UV/Vis and FL spectra of **9c** with solvent compositions of the THF/H_2O mixture upon excitation at 366 nm (1.0 x 10^{-5} M).

NMR spectra:

4-Fluoro-3,5-bis(3-(trifluoromethyl)phenyl)isoxazole (**3d**)

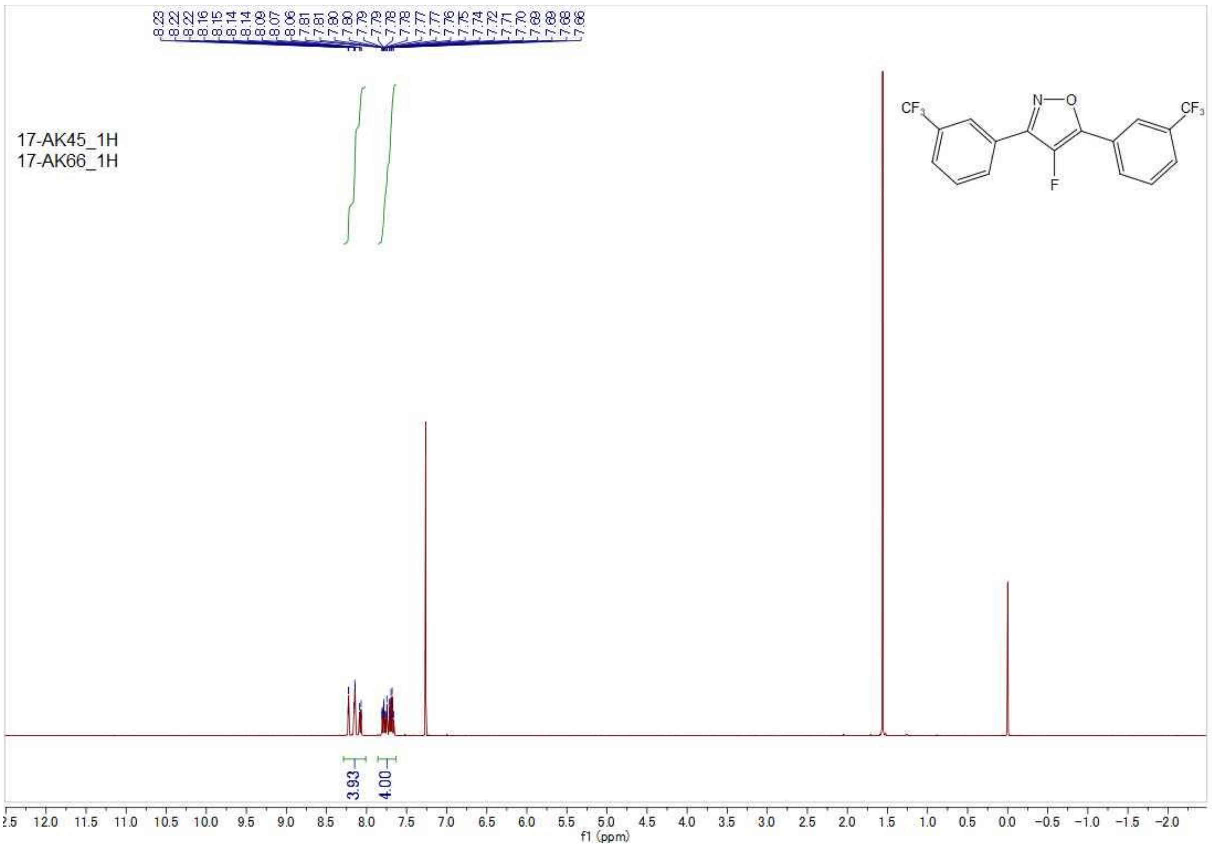

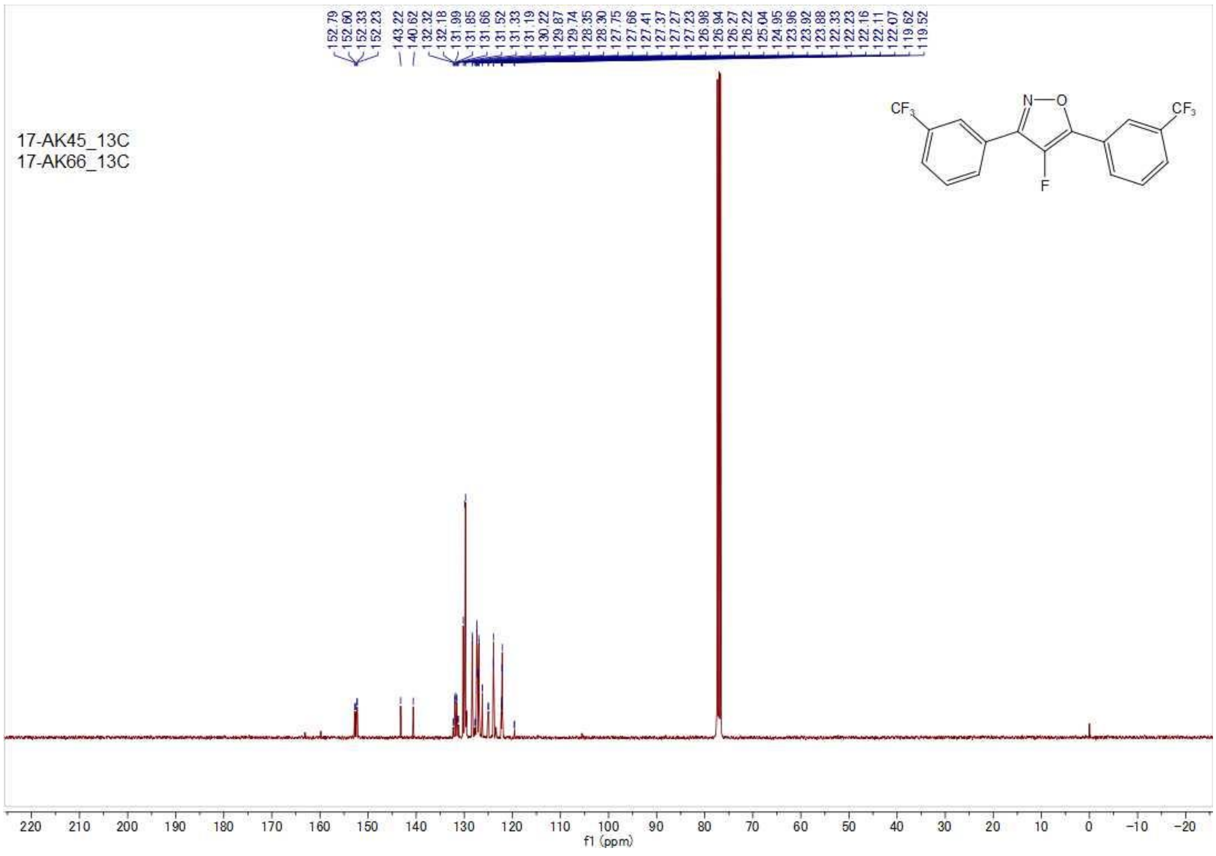

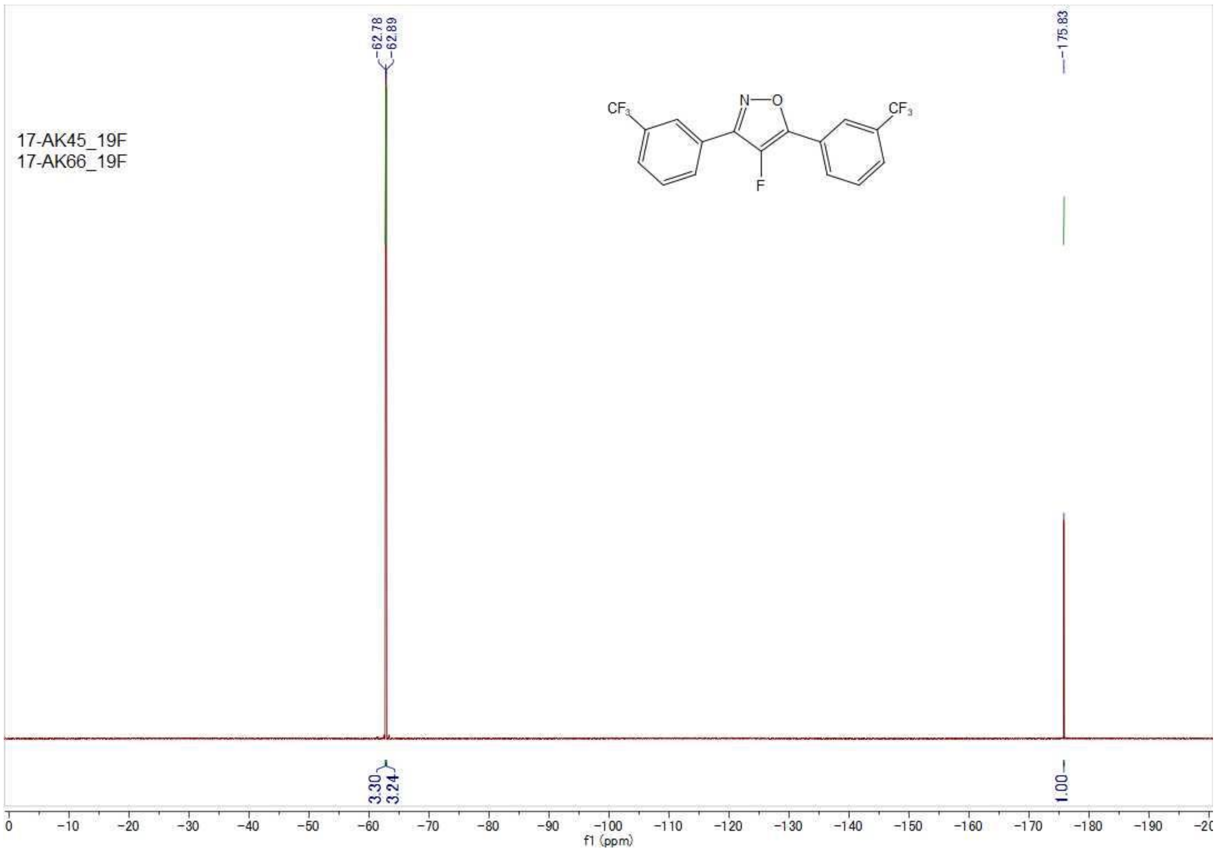

2,2-Difluoro-4,6-diphenyl-2H-1,3,2λ^4-oxazaborinine (**6a**)

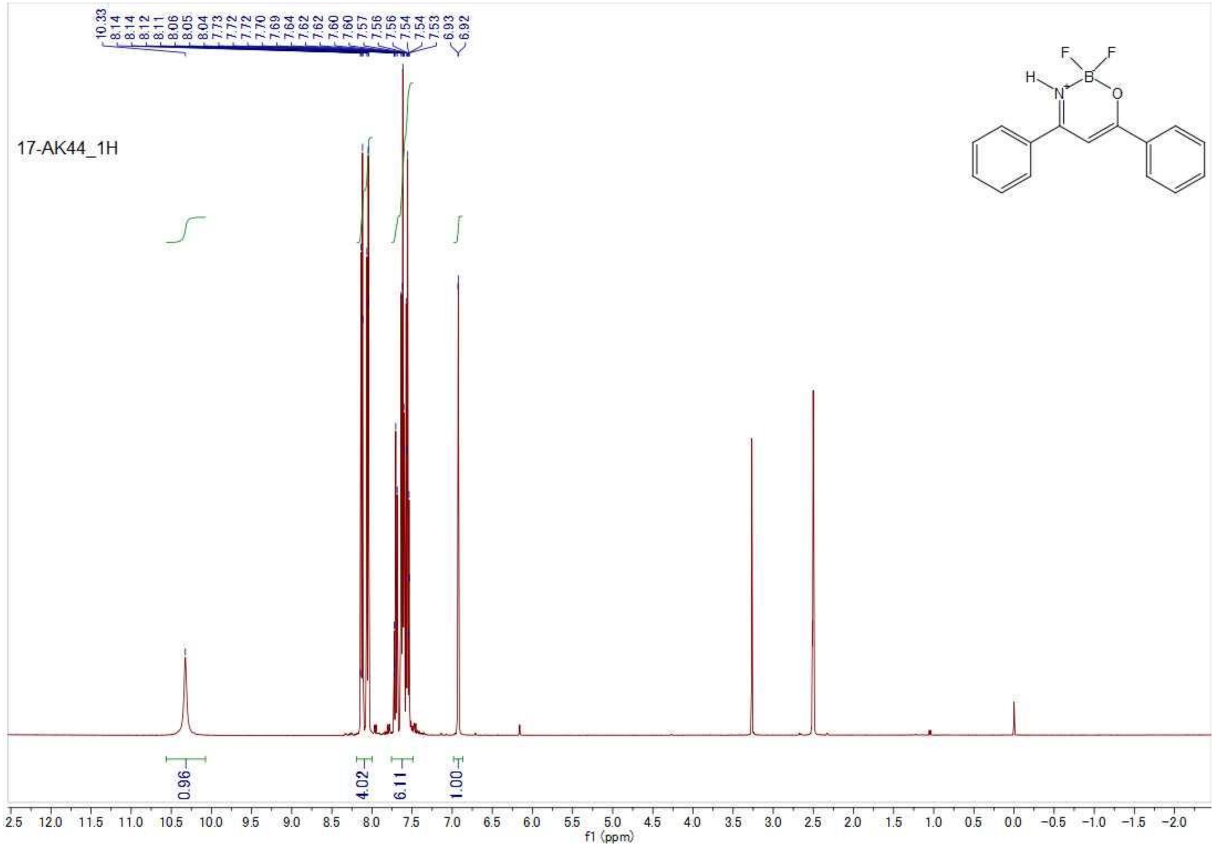

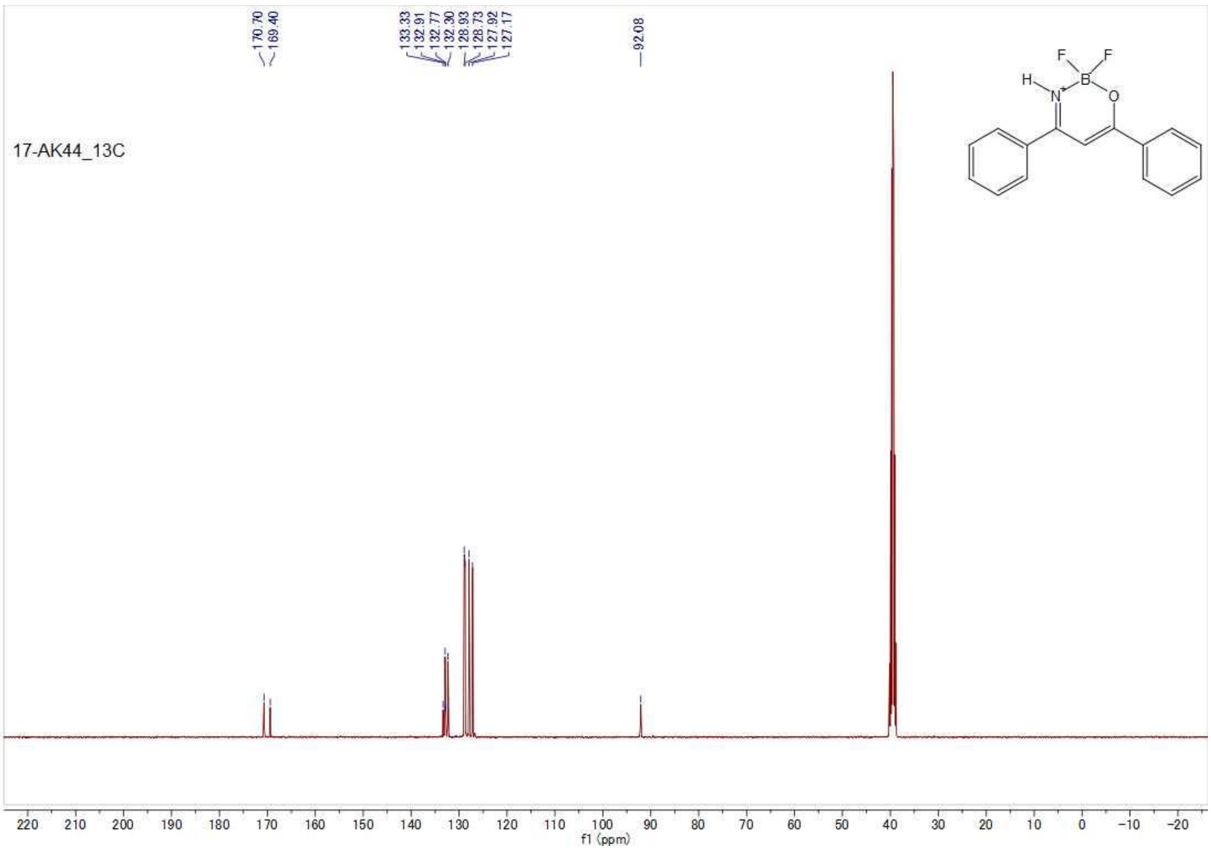
170.70
169.40
133.33
132.91
132.77
132.30
128.93
128.73
127.92
127.17
92.08
17-AK44_13C
F
F
H
N
B
O
220 210 200 190 180 170 160 150 140 130 120 110 100 90 80 70 60 50 40 30 20 10 0 −10 −20
f1 (ppm)

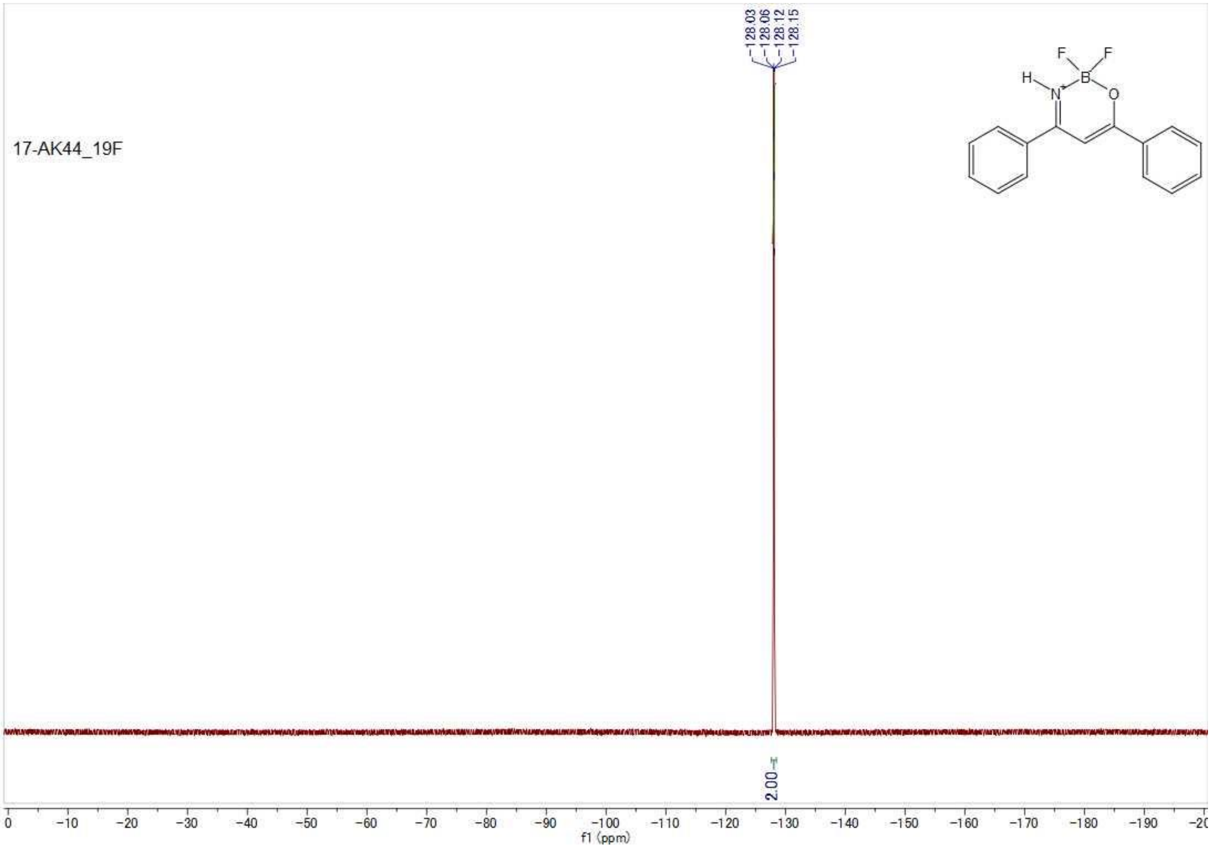
-128.03
-128.06
-128.12
-128.15
17-AK44_19F
F
F
H
N
B
O
2.00
0 −10 −20 −30 −40 −50 −60 −70 −80 −90 −100 −110 −120 −130 −140 −150 −160 −170 −180 −190
f1 (ppm)

2,2-Difluoro-4,6-bis(4-methoxyphenyl)-2*H*-1,3,2λ[4]-oxazaborinine (**6b**)

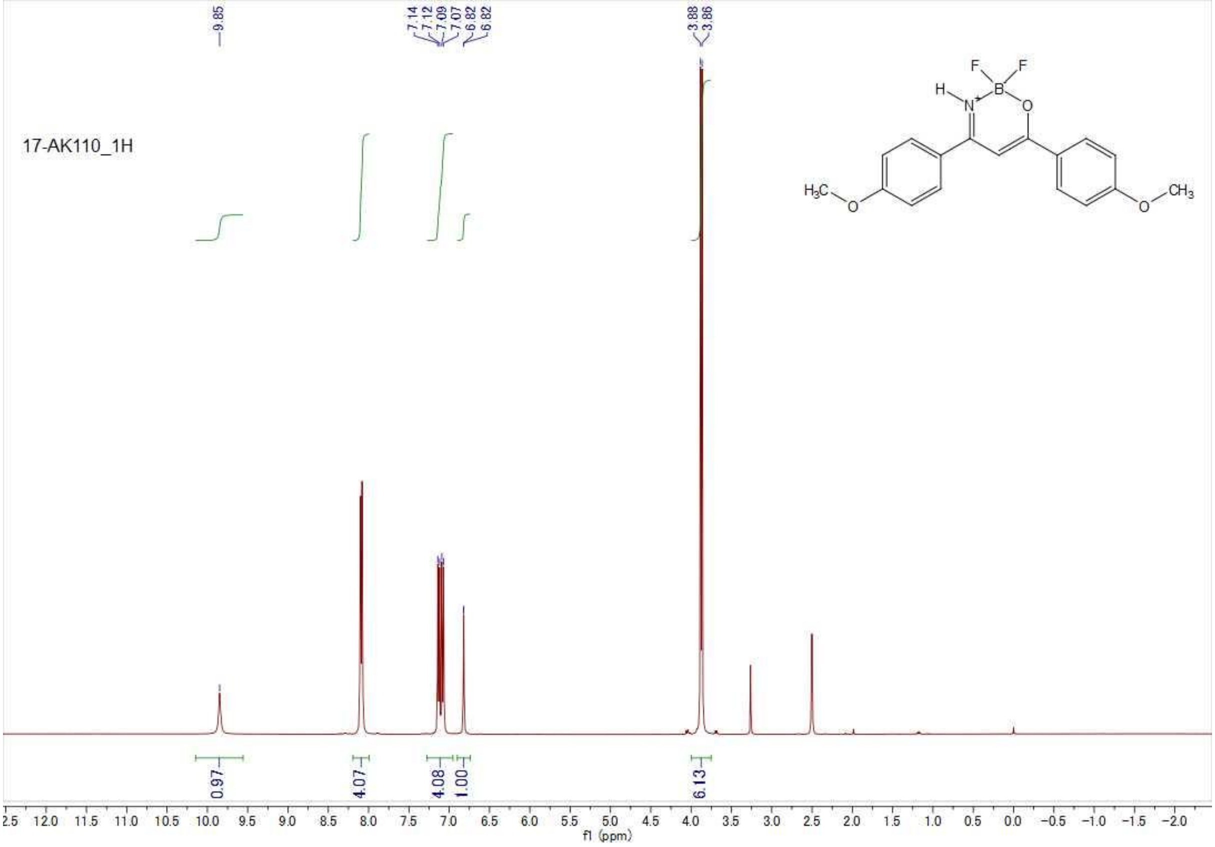

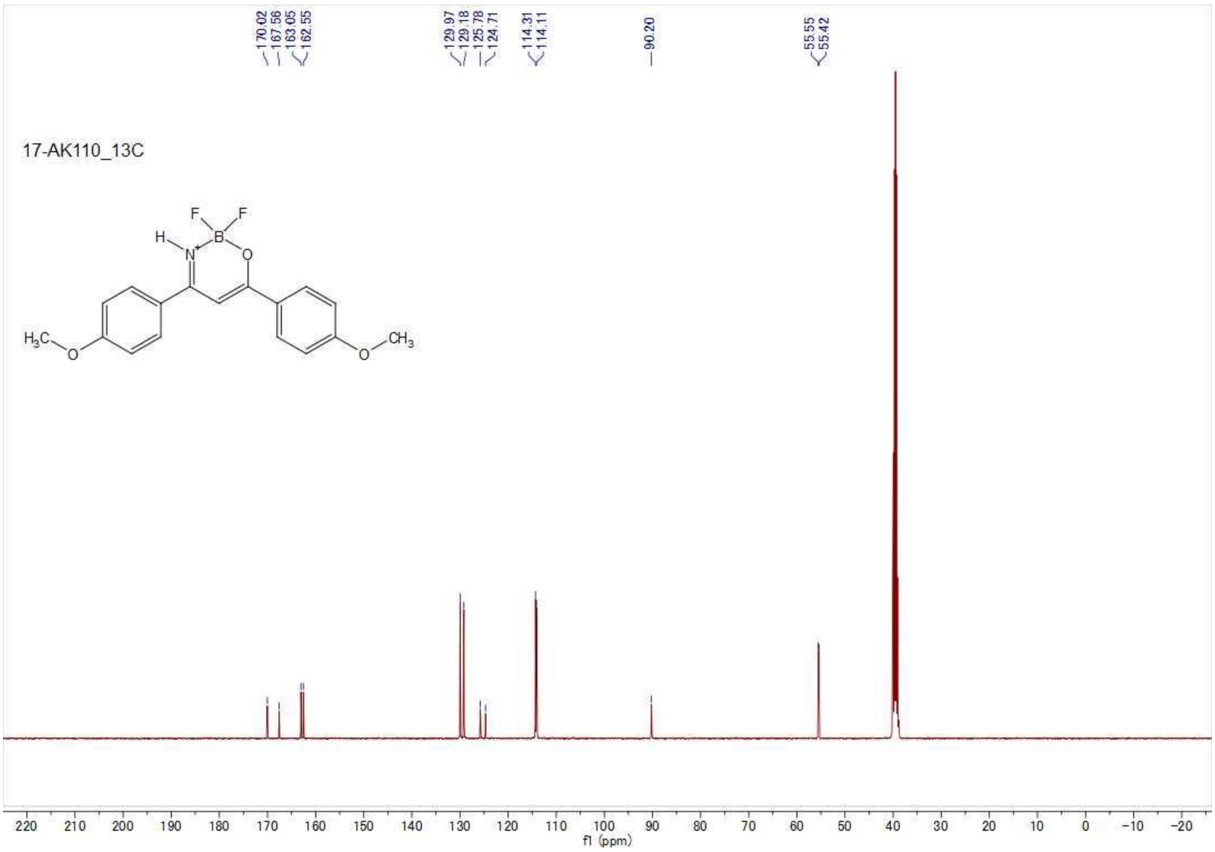

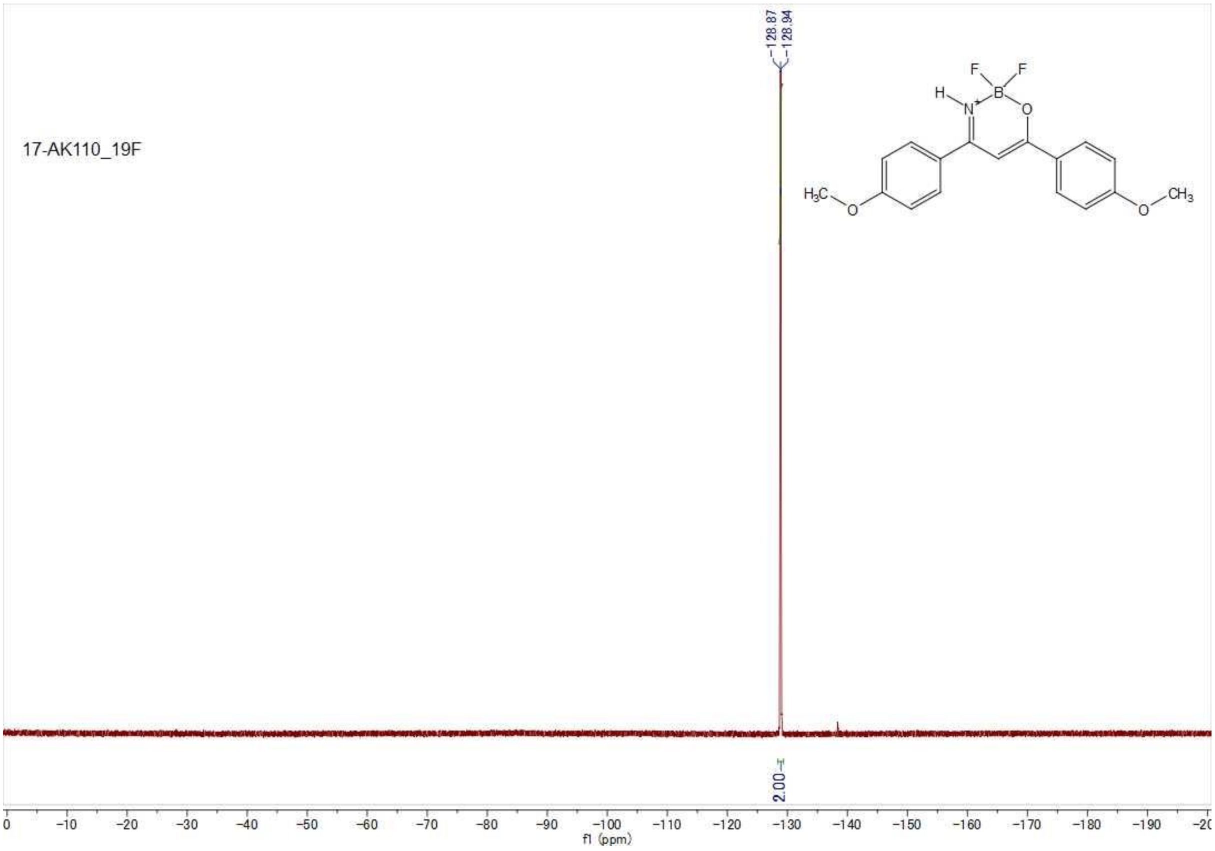

2,2-Difluoro-4,6-bis(4-(trifluoromethyl)phenyl)-2H-1,3,2λ^4-oxazaborinine (**6c**)

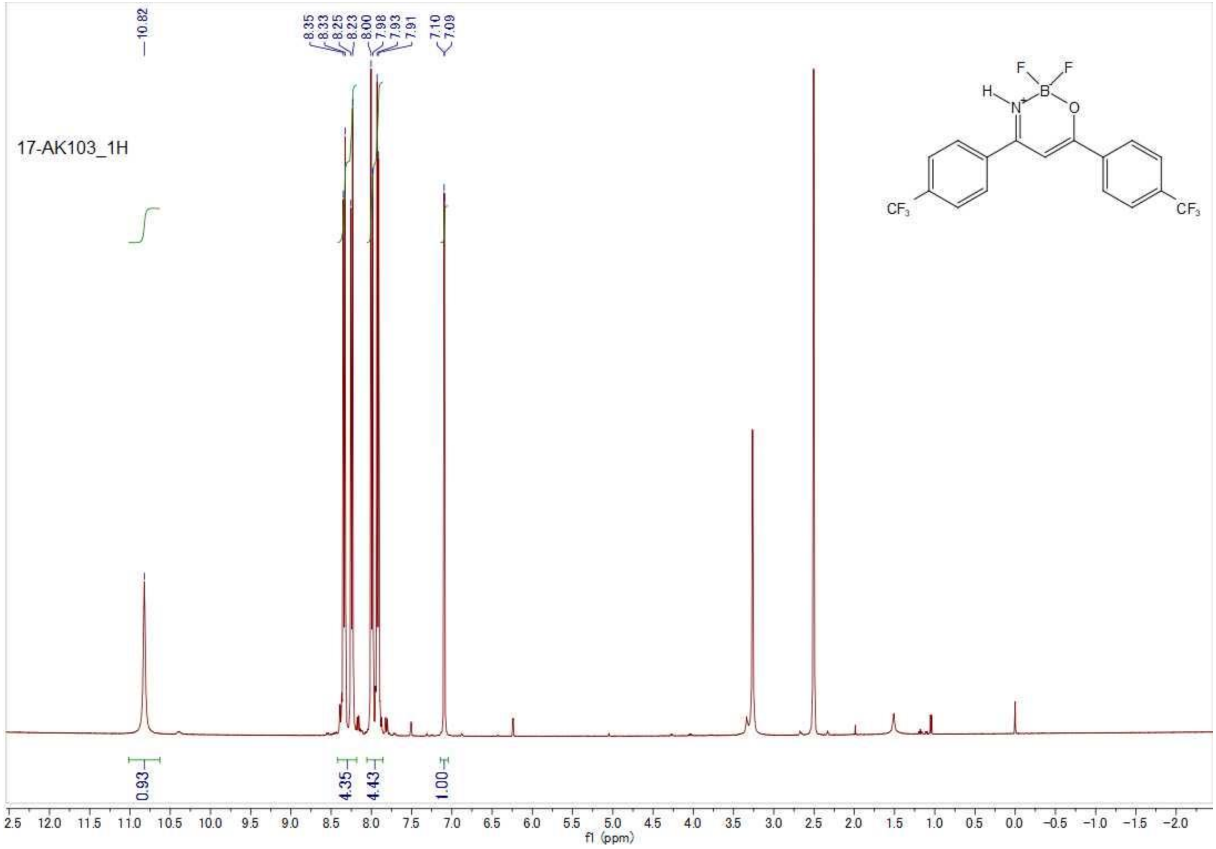

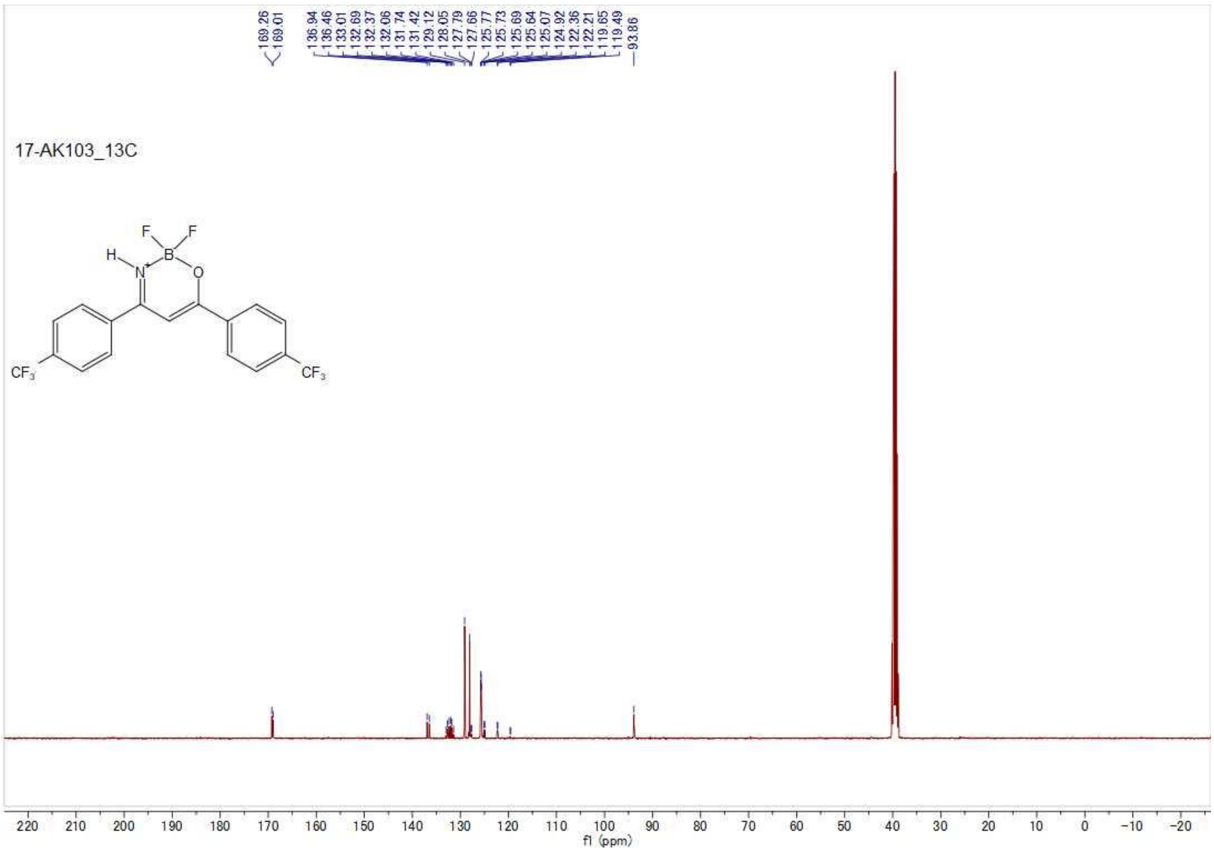
169.26
169.01
136.94
136.46
133.01
132.69
132.37
132.06
131.74
131.42
129.12
128.05
127.79
127.66
125.77
125.73
125.69
125.64
125.07
124.92
122.36
122.21
119.65
119.49
93.86
17-AK103_13C
F
F
H
N
B
O
CF3
CF3
220 210 200 190 180 170 160 150 140 130 120 110 100 90 80 70 60 50 40 30 20 10 0 −10 −20
f1 (ppm)

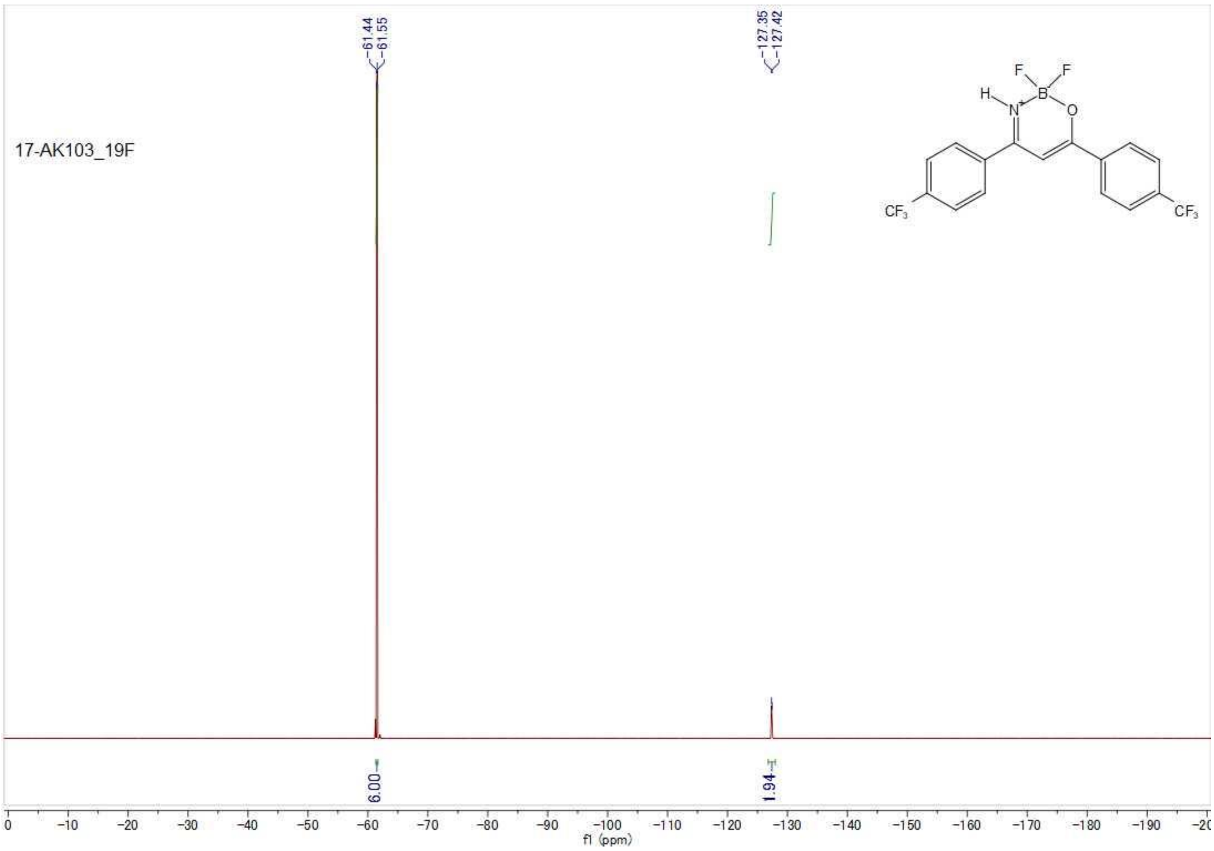
-61.44
-61.55
-127.35
-127.42
17-AK103_19F
F
F
H
N
B
O
CF3
CF3
6.00
1.94
0 −10 −20 −30 −40 −50 −60 −70 −80 −90 −100 −110 −120 −130 −140 −150 −160 −170 −180 −190 −20
f1 (ppm)

3-Amino-2-fluoro-1,3-diphenylprop-2-en-1-one (**8a**)

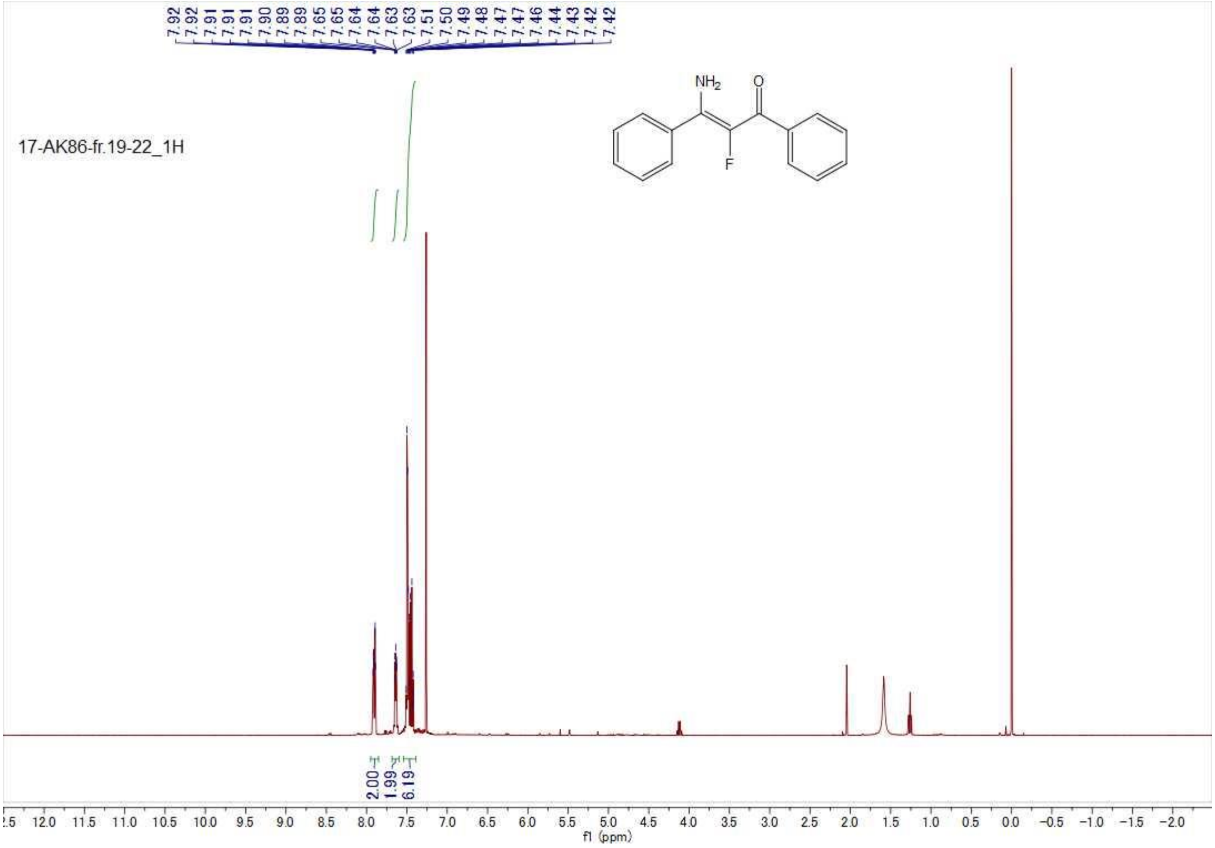

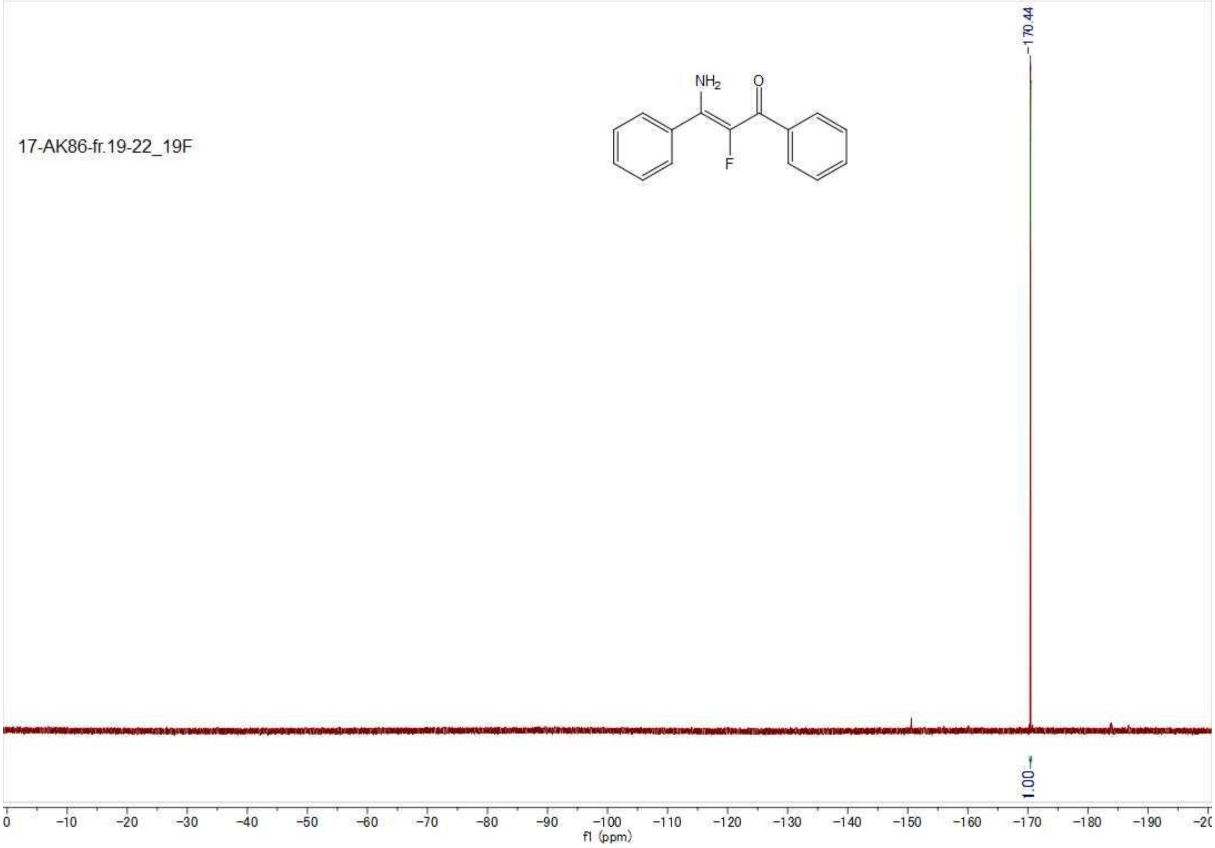

3-Amino-2-fluoro-1,3-bis(4-methoxyphenyl)prop-2-en-1-one (**8b**)

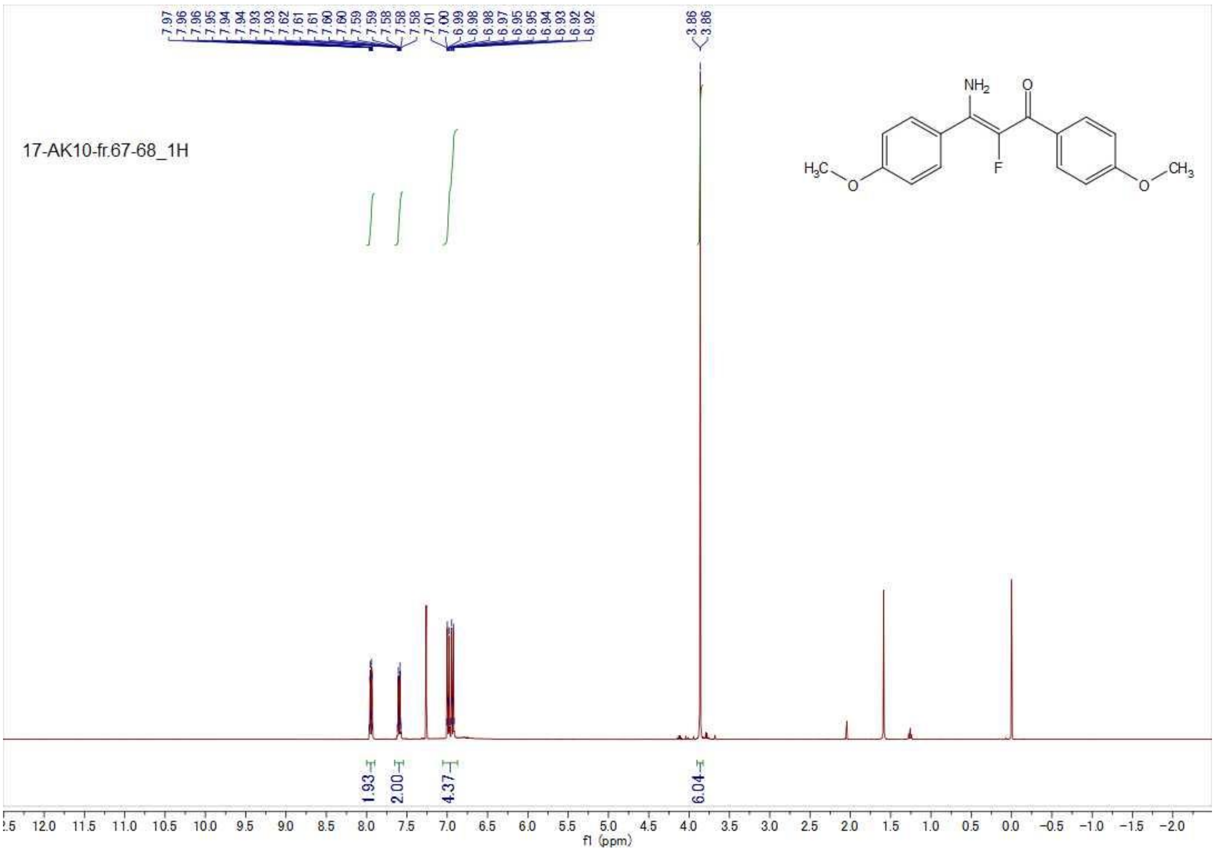

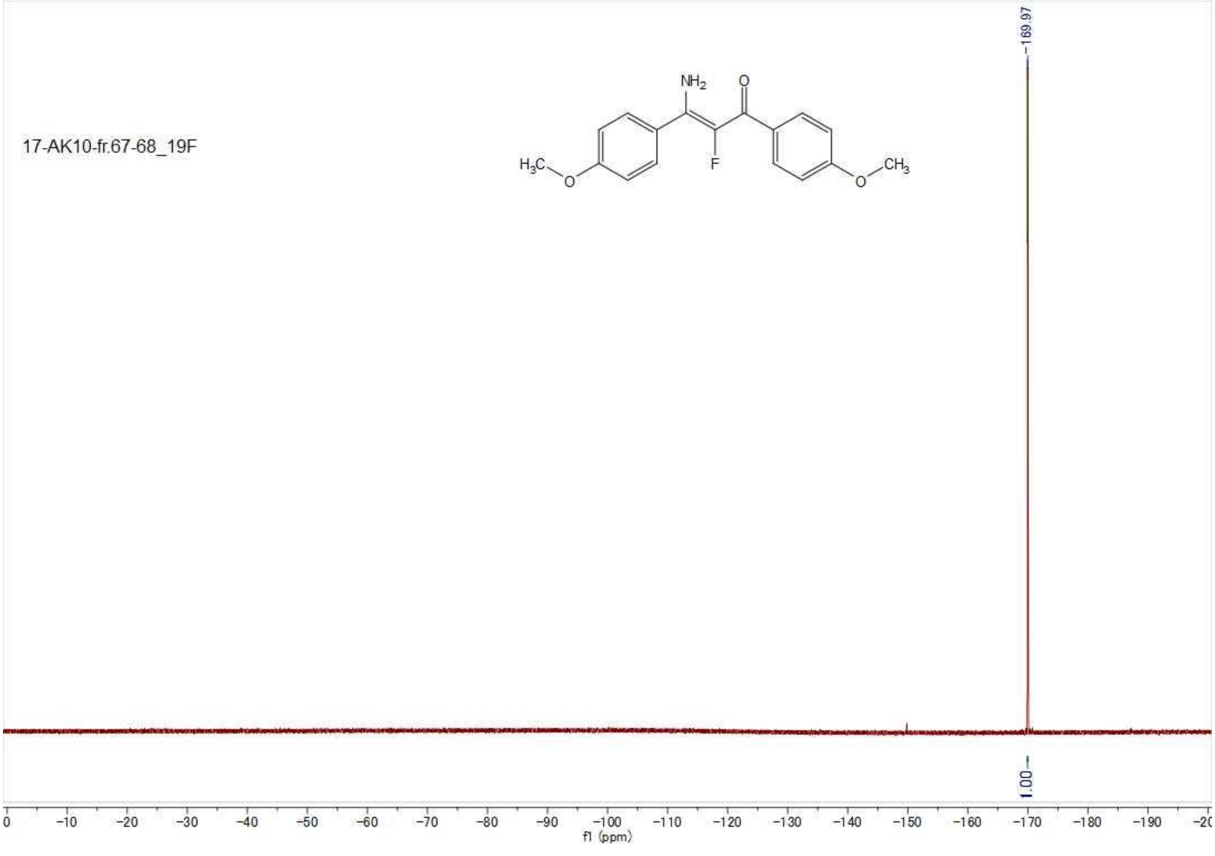

3-Amino-2-fluoro-1,3-bis(4-(trifluoromethyl)phenyl)prop-2-en-1-one (**8c**)

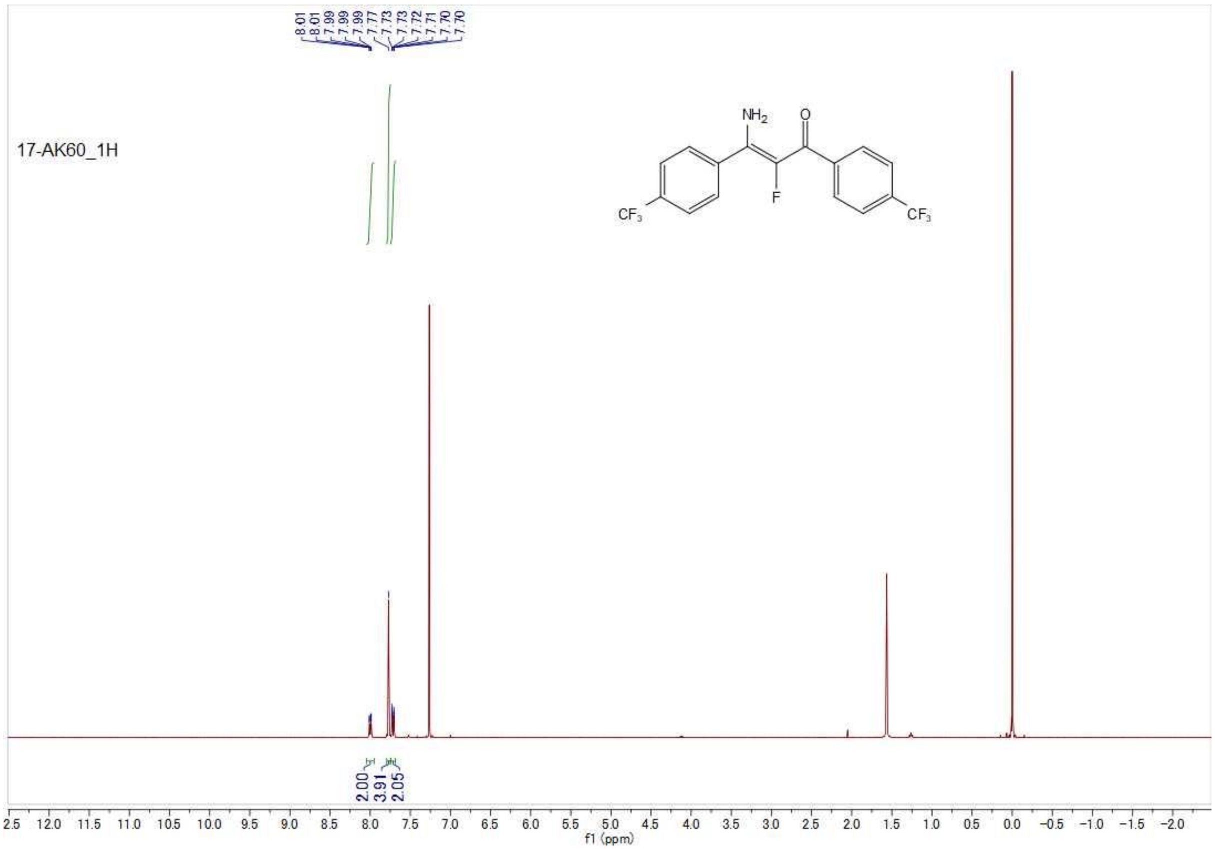

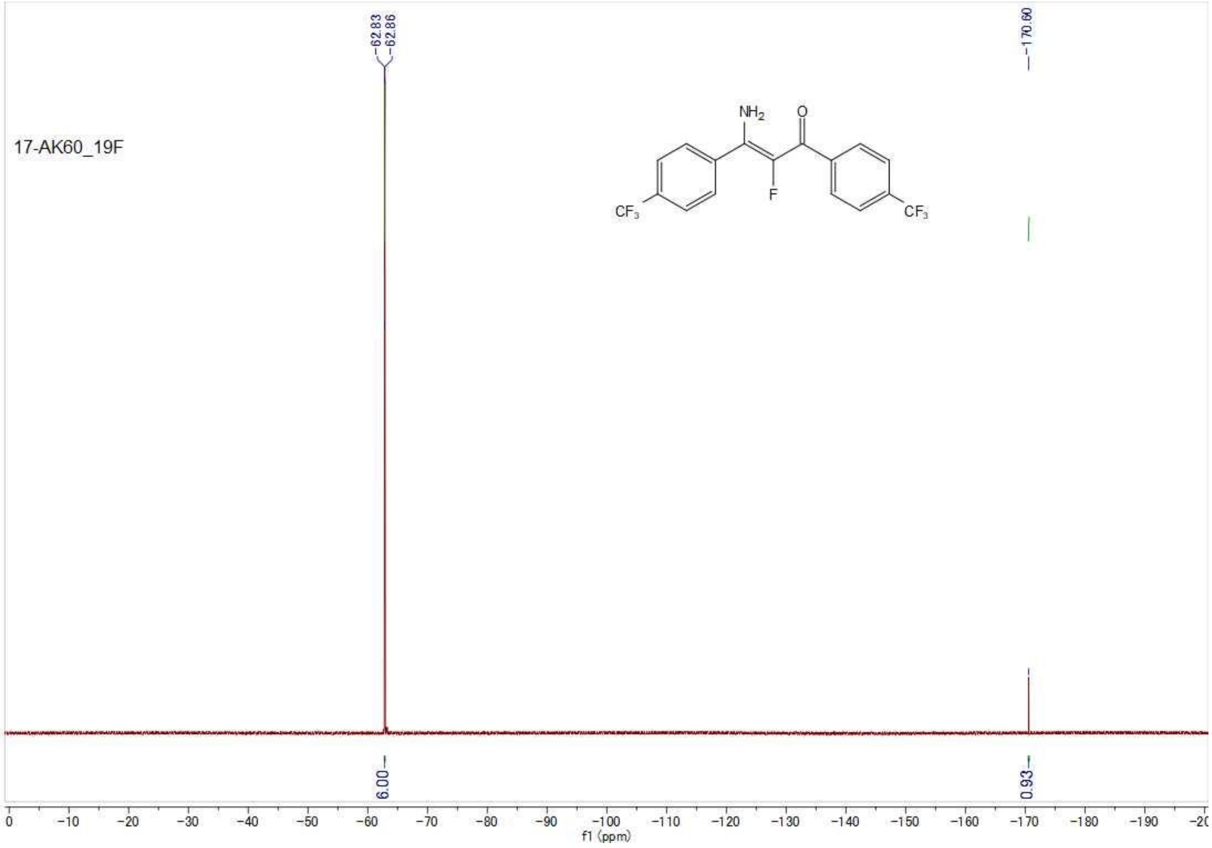

2,2,5-Trifluoro-4,6-diphenyl-2*H*-1,3,2λ^4-oxazaborinine (**9a**)

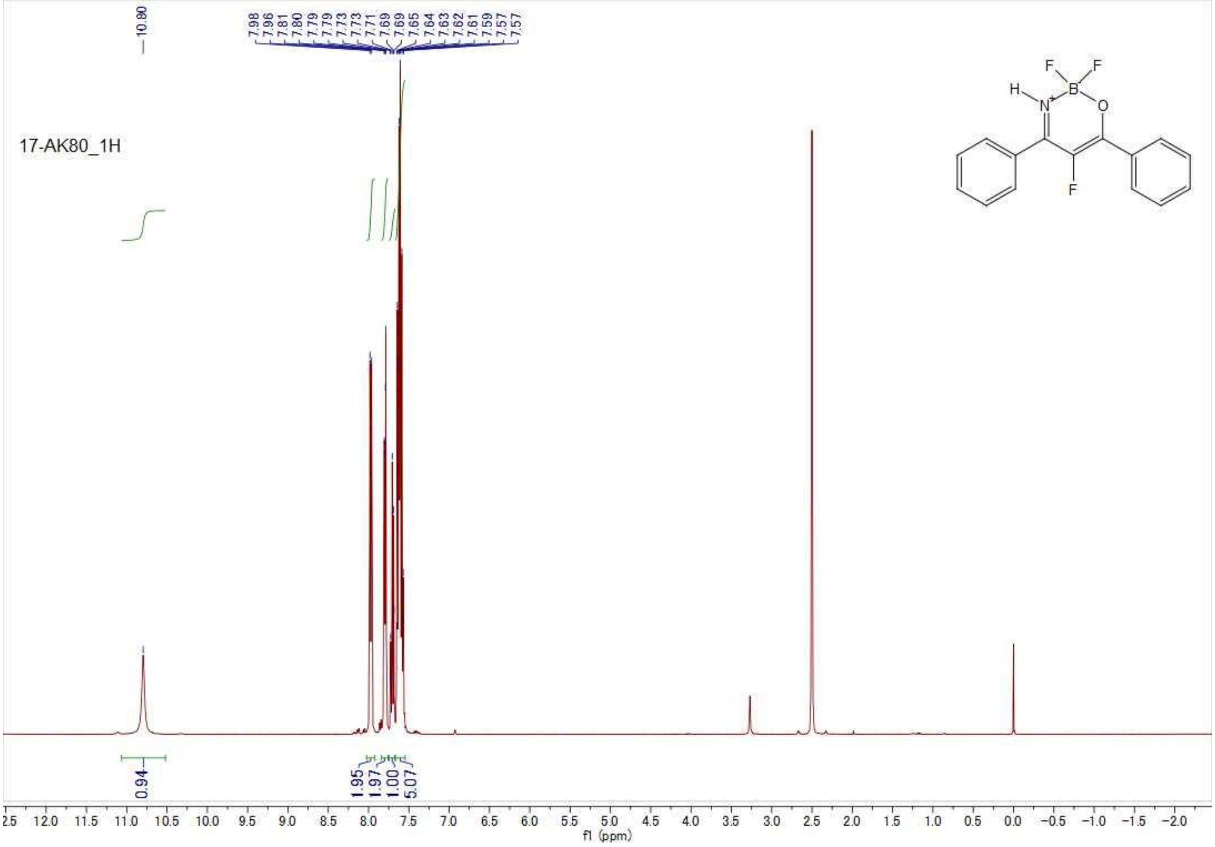

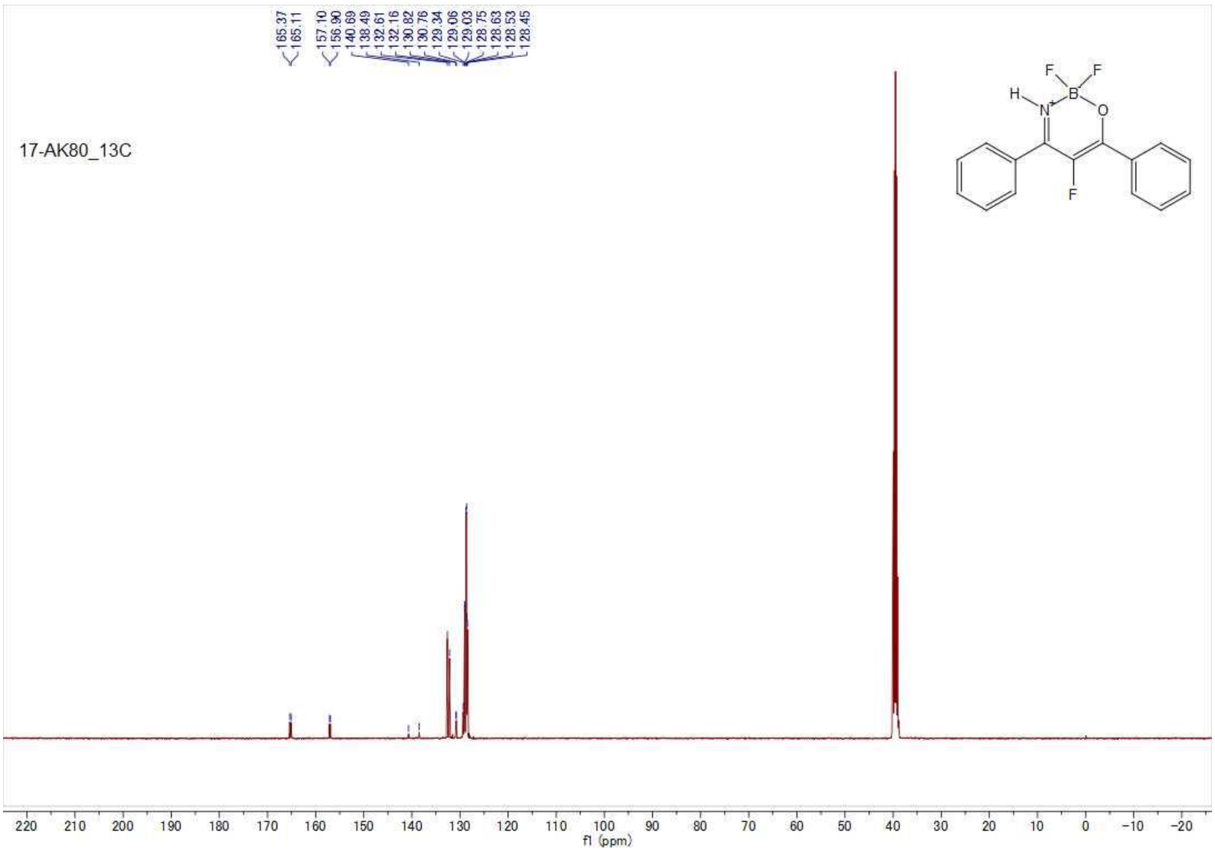

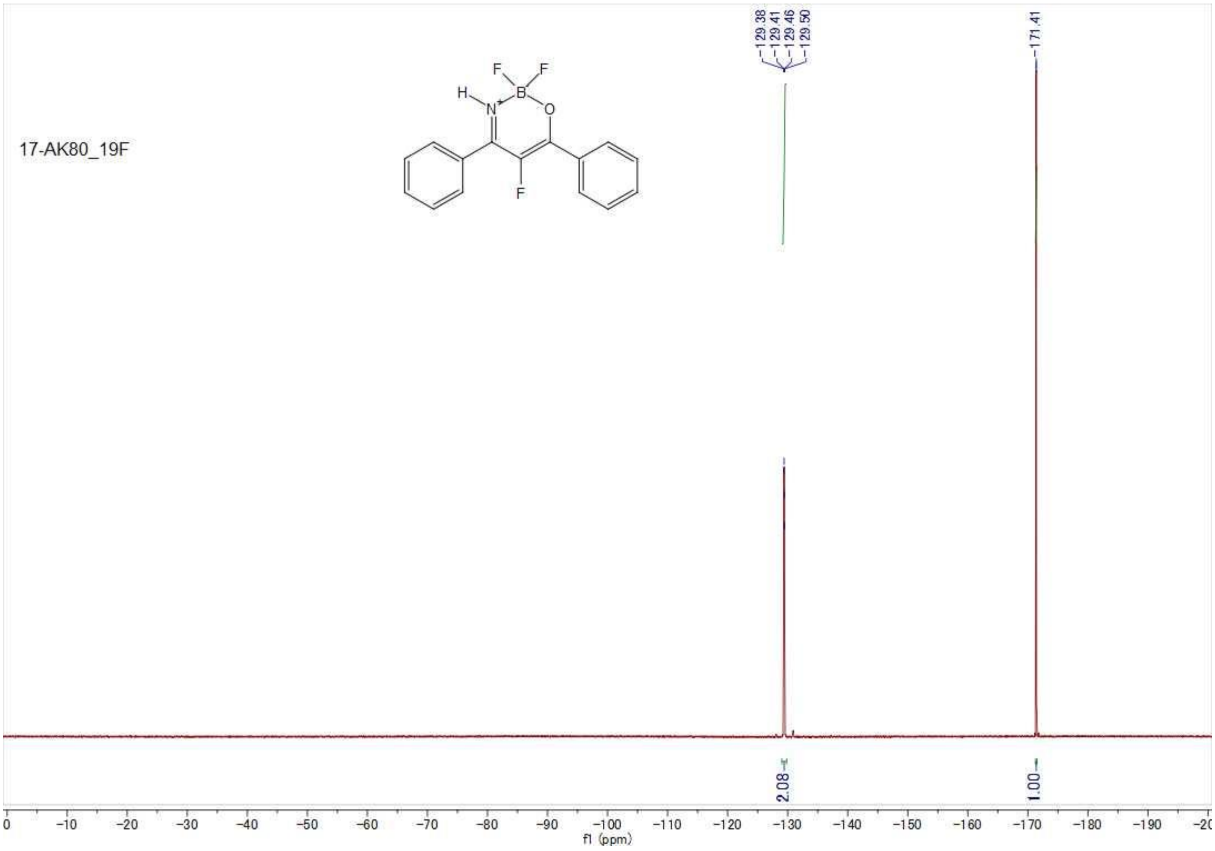

2,2,5-Trifluoro-4,6-bis(4-methoxyphenyl)-2*H*-1,3,2λ^4-oxazaborinine (**9b**)

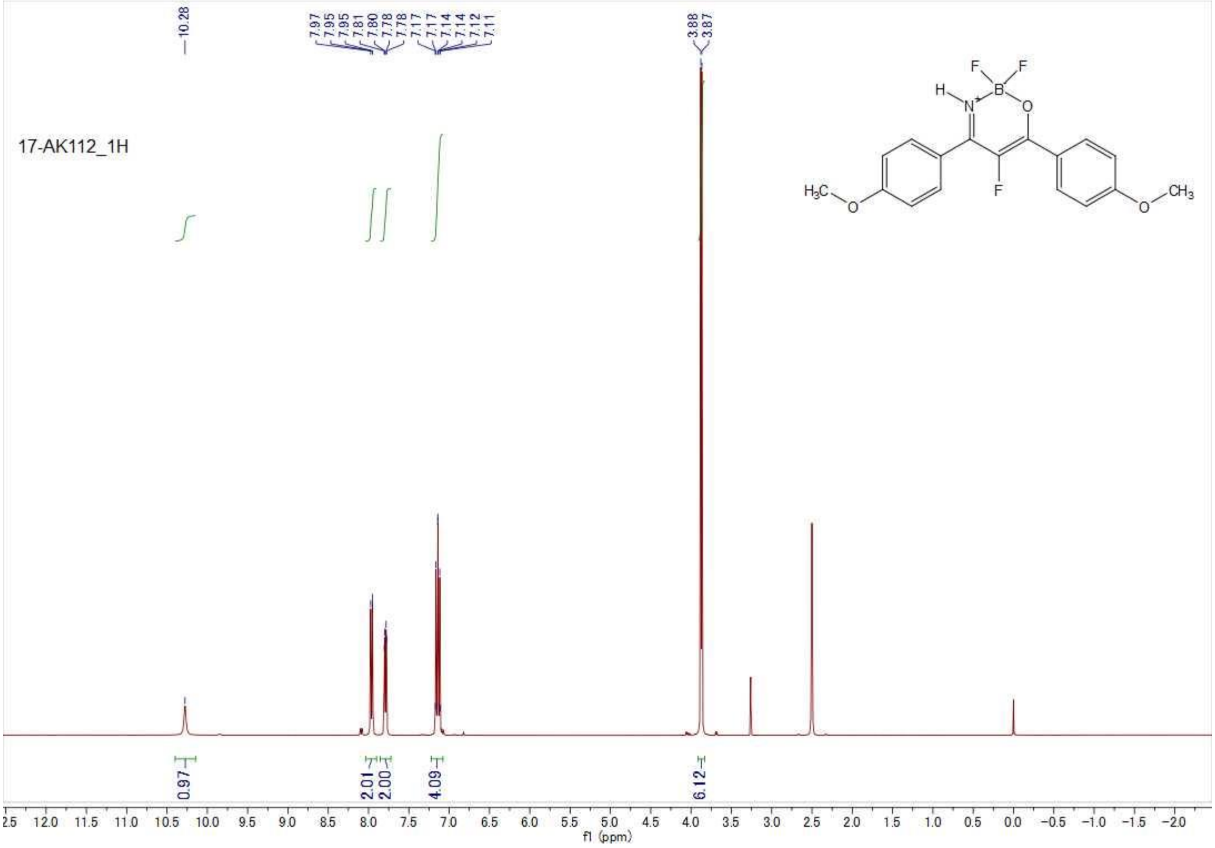

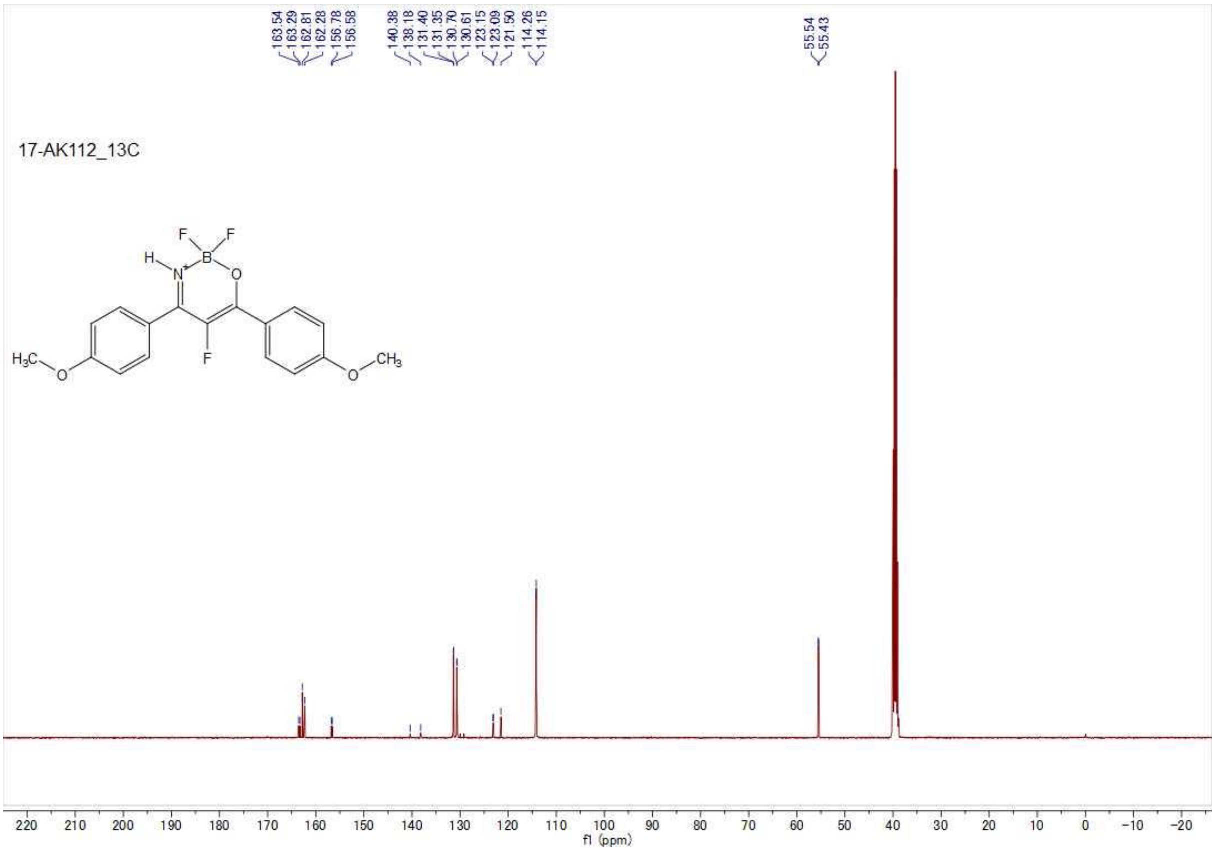
17-AK112_13C
163.54
163.29
162.81
162.28
156.78
156.58
140.38
138.18
131.40
131.35
130.70
130.61
123.15
123.09
121.50
114.26
114.15
55.54
55.43
f1 (ppm)

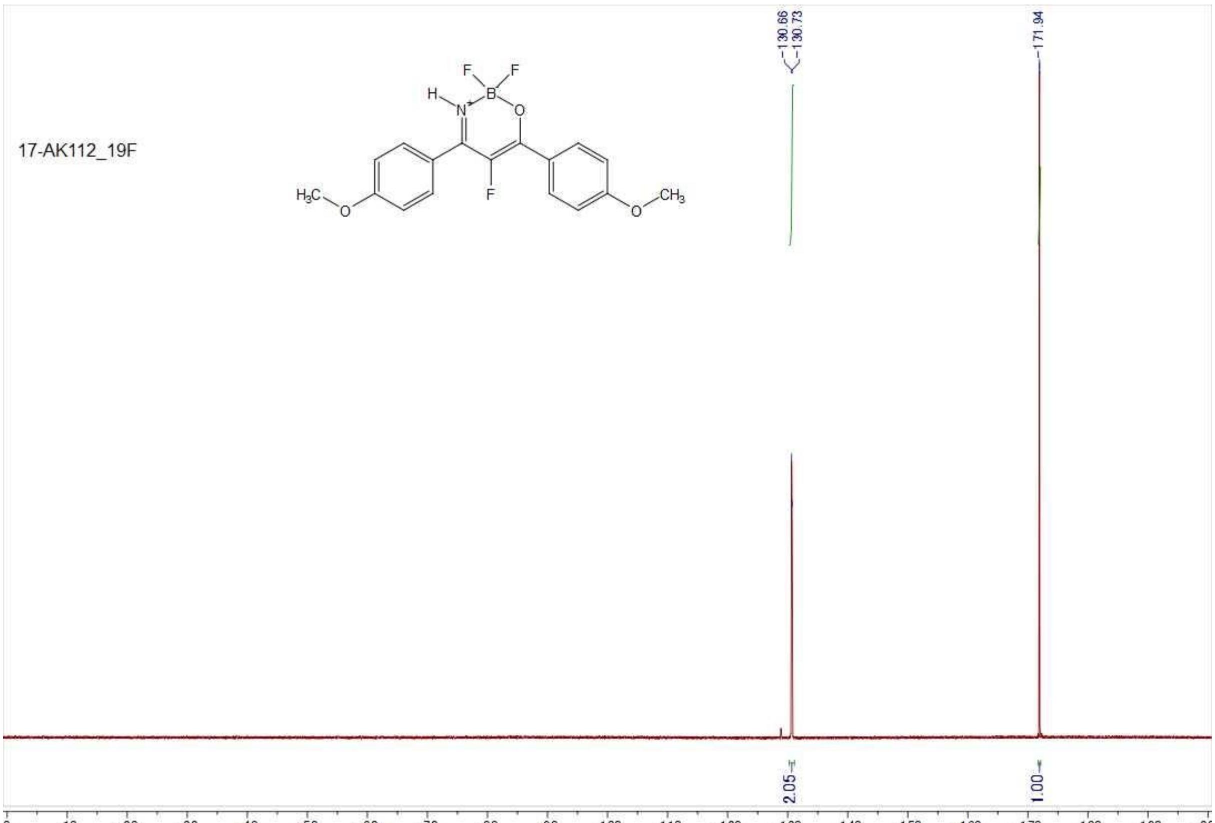
17-AK112_19F
-130.66
-130.73
-171.94
2.05
1.00
f1 (ppm)

2,2,5-Trifluoro-4,6-bis(4-(trifluoromethyl)phenyl)-2*H*-1,3,2λ[4]-oxazaborinine (**9c**)

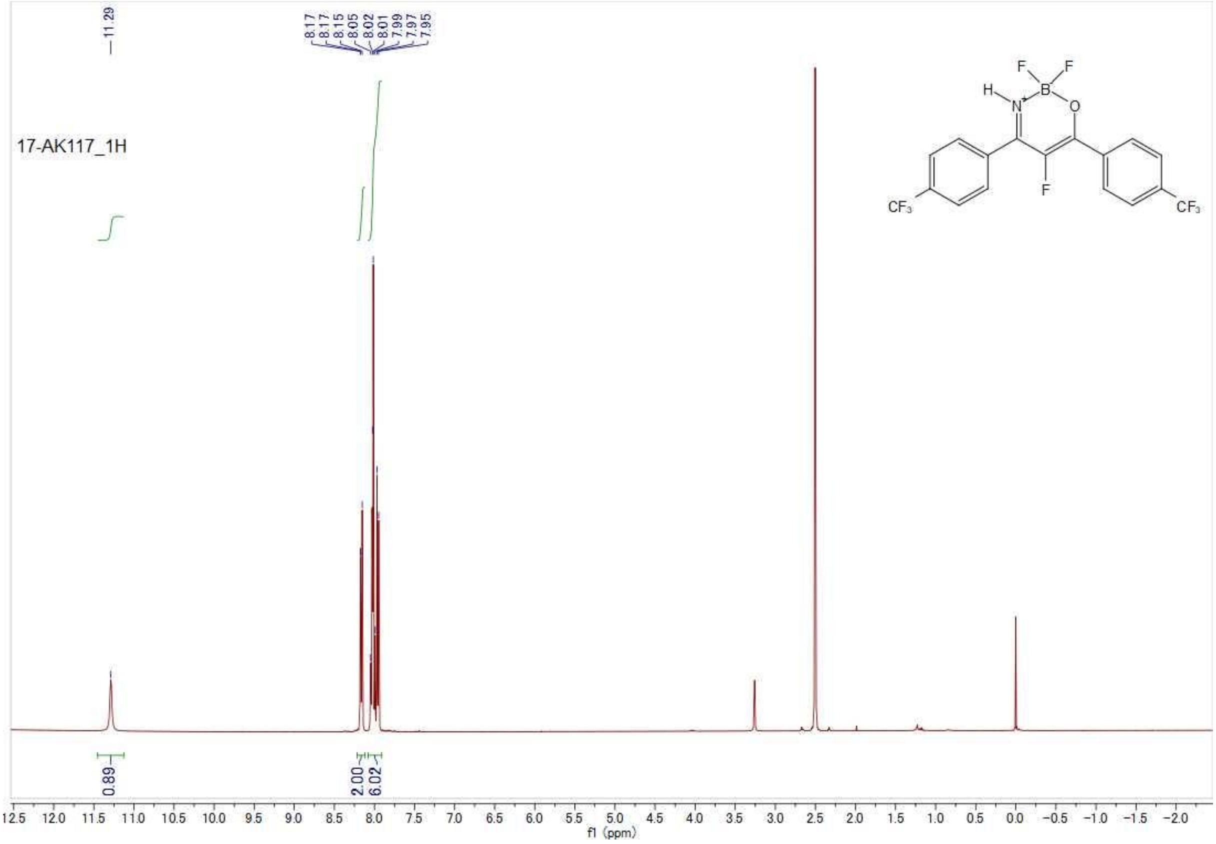

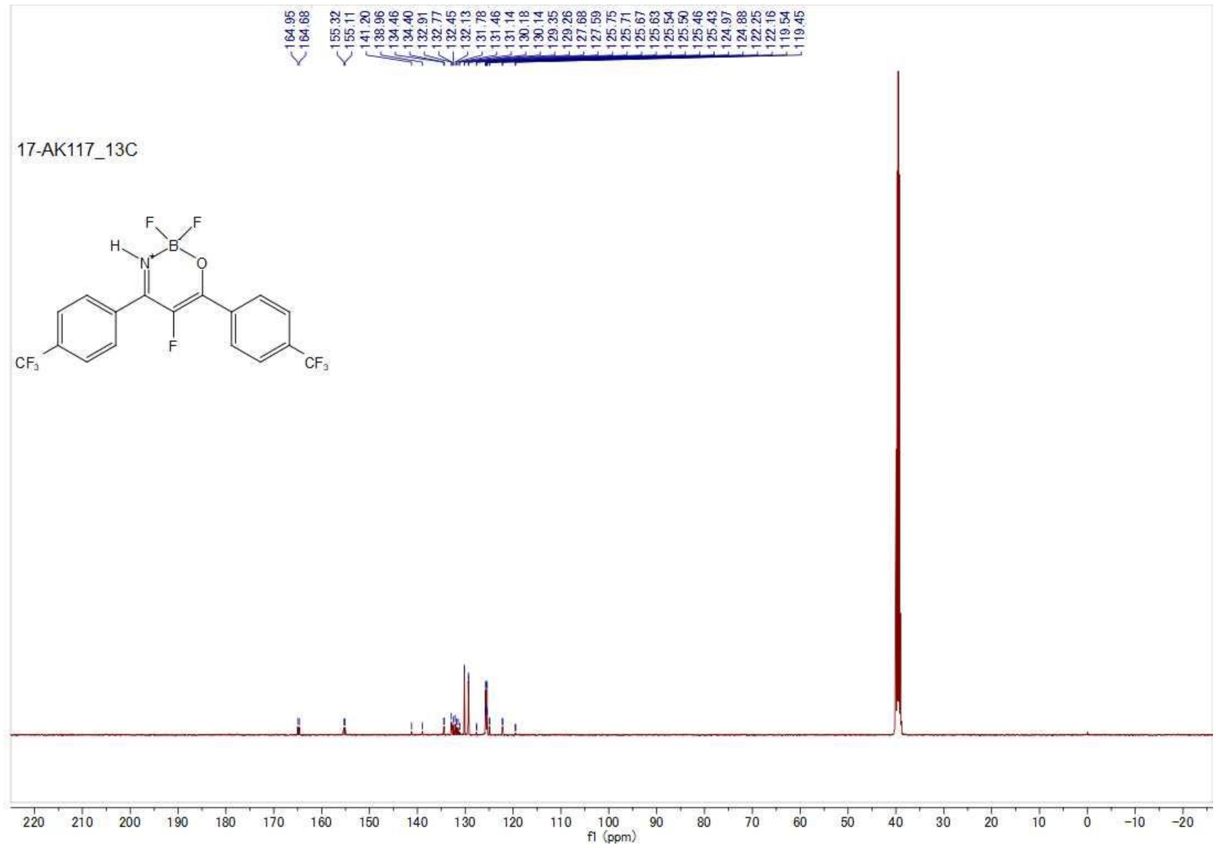

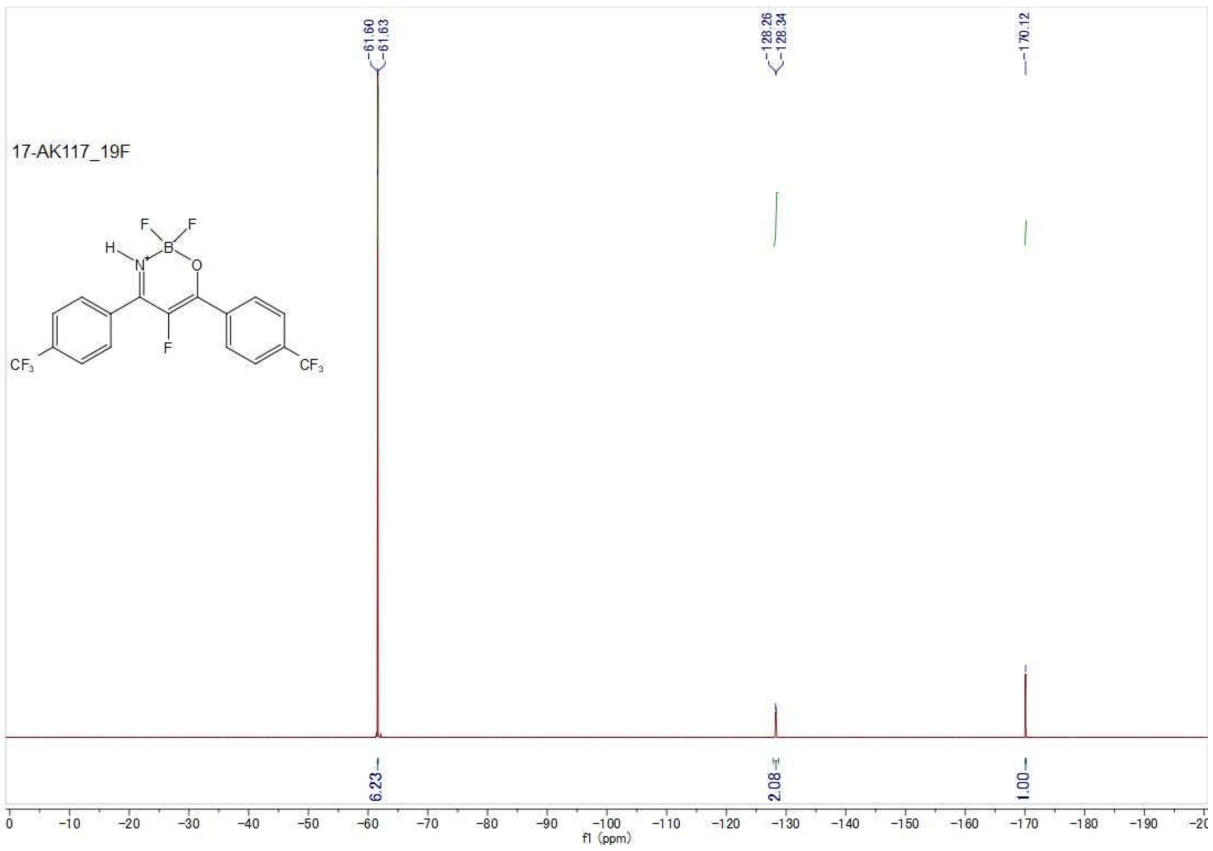

17-AK117_19F
-61.60
-61.63
-128.26
-128.34
-170.12
6.23
2.08
1.00
f1 (ppm)
